# Archaeogenomic Analysis of Nineteenth‐Century Burials at Saint Mary's Basilica: An Intersectional Analysis of Religion, Race, and Migration

**DOI:** 10.1002/ajpa.70110

**Published:** 2025-08-14

**Authors:** Raquel E. Fleskes, Horvey M. Palacios, Hannah Budner, Dana Kollmann, Cassandra Newby‐Alexander, Reed Harder, Deborah A. Bolnick, Marcus Pollard, Paige Pollard, Theodore G. Schurr, David A. Brown

**Affiliations:** ^1^ Department of Anthropology Dartmouth College Hanover New Hampshire USA; ^2^ Department of Anthropology University of Connecticut Storrs Connecticut USA; ^3^ Department of Anthropology The University of Oklahoma Norman Oklahoma USA; ^4^ Department of Neurology University of Pennsylvania Philadelphia Pennsylvania USA; ^5^ Department of Sociology, Anthropology & Criminal Justice Towson University Baltimore Maryland USA; ^6^ Department of History Norfolk State University Norfolk Virginia USA; ^7^ Institute for Systems Genomics University of Connecticut Storrs Connecticut USA; ^8^ Commonwealth Preservation Group Norfolk Virginia USA; ^9^ Department of Anthropology University of Pennsylvania Philadelphia Pennsylvania USA; ^10^ The Fairfield Foundation White Marsh Virginia USA

**Keywords:** ancestry, ancient DNA, bioarchaeology, Catholicism, North America

## Abstract

**Objectives:**

The Basilica of Saint Mary's of the Immaculate Conception in Norfolk, Virginia, is the only predominantly African American basilica in the United States. A community‐based archaeogenomic investigation was carried out at this church to investigate the history of its previous congregants.

**Materials and Methods:**

Five burials were selected for excavation and archaeological analysis. The skeletal remains of these past congregants were assessed to determine age, sex, and preservation status. Ancient DNA was extracted at the University of Connecticut's ancient DNA laboratory.

**Results:**

Five burials excavated at the basilica yielded four sets of human remains of variable preservation and sex. Interment location, style, and positioning of the burials suggested that they belong to the earlier Saint Patrick's church (AD 1790s to 1856), which once stood adjacent to the current basilica. Osteological analyses indicated the presence of four adults and one infant. Ancient DNA results for three of the adults indicated that they were genetically affiliated with contemporary populations in Europe, specifically the United Kingdom, France, and Spain. Interestingly, the autosomal and uniparental lineages of two adults showed connections to Spanish populations, with one having a unique L3f1b mitochondrial DNA haplotype tracing back to northern Spain.

**Conclusions:**

Using an intersectional theoretical framework grounded in historical research, we assess these findings to understand the lived experiences of these past congregants in the context of religion, race, and migration in early nineteenth‐century Norfolk. Overall, this study highlights the value of an interdisciplinary archaeogenetic approach in exploring the intersectional lives of historic populations.

**Summary**

- Archaeogenomic analysis reveals European genomic ancestry, with a unique L3f1b mitochondrial haplotype tracing to northern Spain being identified.
- An intersectional approach highlights the role of religion, race, and gender in shaping lived experiences.

## 1 Introduction

Catholicism in the United States during the eighteenth and nineteenth century was predominantly viewed as a minority religious denomination. Its practitioners were largely European migrants seeking religious freedom but also included both enslaved and free African Americans in North America (Fogarty 2001). Some of these persons settled in Norfolk, Virginia, a port city on the eastern coast of the present‐day United States. Early Catholic life in Norfolk formed, in part, around Saint Patrick's—the city's first Catholic church (Parramore et al. 2000; Fogarty 2001). Built in the 1790s, Saint Patrick's served both Black and white Catholic communities in Norfolk until it was burned down in 1856 (Yarsinske 1999; Fogarty 2001; Pollard 2019). Today, the Basilica of Saint Mary's of the Immaculate Conception sits adjacent to the foundations of Saint Patrick's, carrying the legacy of early Catholics in Virginia into the present day (Pollard 2019).

When burials were discovered during renovations to Saint Mary's Basilica in 2019, it offered an opportunity to more deeply investigate this community's multi‐layered history. Today, Saint Mary's stands as the only predominantly African American basilica in the United States (Pollard 2019). The contemporary congregation formed in the 1960s after a merger of the congregations of St. Mary's, a historically White Catholic church, and Saint Joseph's, a historically Black Catholic church, and reflects the demographics of the surrounding community today (Rose 2007).

After the burials were discovered, congregation members expressed their interest in learning more about those buried beneath the floor of Saint Mary's to explore the church's deeper history and their spiritual connections to these earlier members of the congregation. Thus, to learn more about these histories, a community‐centered archaeogenomic investigation was carried out using archaeological, osteological, and ancient DNA (aDNA) methods. Here, we report the results of these analyses and apply an intersectional theoretical framework to evaluate migration, religion, and race in nineteenth‐century Norfolk.

### 1.1 Historical Context

During the colonial era, European imperialist desires for religious, wealth, and racial domination differentially shaped colonial settlements in the Americas (Kupperman 2007; Richter 2011). Early Spanish and French imperial efforts were driven, in part, by a moral motivation to convert Indigenous communities in the Americas and the Caribbean to Catholicism (Greer and Mills 2017). English colonial settlements followed suit in an effort to curb the growing influence of these competing imperial interests (Richter 2011). For example, Virginia was founded as an English colony in the early seventeenth century on lands inhabited by the Powhatan, Chickahominy, Mattaponi, Pamunkey, Monacan, Nansemond, and other Indigenous tribes in order to limit Spanish expansion in Florida and the Carolinas, provide natural resources to the Crown, and establish the Church of England in the “New World” (Kupperman 2007).

The neighboring colony of Maryland was originally chartered a few decades later as a sanctuary for English Catholics facing persecution from Protestants in England, thereby establishing itself as a hub for Catholic migration in the region (Andrews 1936). However, shifting attitudes toward religious tolerance significantly impacted Catholic settlement patterns. The Maryland Protestant Revolution of 1689 weakened Catholic authority in the colony, diminishing its status as a stronghold for English Catholic life (Andrews 1936). A century later, the Virginia Statute of Religious Freedom was passed, institutionalizing tolerance of all religious practices in the state (Peterson and Vaughan 1988). This disestablishment of state‐sponsored religion encouraged an increasing number of Catholics to relocate to Virginia, particularly to the port city of Norfolk, which had become one of the state's most populous cities (Fogarty 2001).

Catholic migration to Norfolk was also connected to other diasporas in the Atlantic world, including those stemming from the French and Haitian Revolutions (Yarsinske 1999; Parramore et al. 2000). Anti‐Catholic sentiments during the French Revolution prompted some French Catholics to seek refuge in the colonies, with some settling in Norfolk (Parramore et al. 2000). The city's port also became a gateway for thousands of displaced refugees fleeing the Haitian Revolution of 1791 (Bell 2020), the first successful uprising by enslaved persons to result in the establishment of a free and independent Black state in the Americas (Alexander 2022; Garrigus 2023). Among these migrants were white planters and enslaved and free Africans (Sidbury 1997). This influx of people and their accompanying revolutionary ideals fueled fears of an insurrection in Virginia, whose economy was founded on the oppression and enslavement of persons of African descent (Sidbury 1997; Geggus 2001).

Saint Patrick's Catholic Church was formed shortly after this period and represented the only Catholic Church in Norfolk until the late 20th century (Fogarty 2001). In 1794, the Trustees of the Roman Catholic Society of Norfolk Borough purchased a plot at the corner of Chapel and Holt Streets, where Saint Patrick's was—and Saint Mary's now is—located (Fogarty 2001; Pollard 2019). The original wood‐framed chapel was built in 1802–1803 and overseen by French emigrés, Father James Lucas and Father Lacy (Fogarty 2001). It subsequently went through a number of structural changes, including its replacement with a small brick church in 1831 and its expansion in 1842 (Fogarty 2001; Pollard 2019).

Black Catholics also worshiped at Saint Patrick's during this period. Early records indicate that white and Black parishioners participated in combined masses (Fogarty 2001; Pollard 2019). Notes from Father Delaney's tenure in 1824 suggest that, while the number of Black parishioners was relatively small, it was not insignificant (Fogarty 2001).

Between 1785 and 1852, Norfolk's Catholic population grew from about 200 to an estimated 11,000 to 18,000 persons (Fogarty 2001; Pollard 2019). Despite this explosive growth, xenophobic sentiments persisted throughout Virginia, fueled by groups such as the Know‐Nothing Party, an anti‐Catholic, and anti‐immigrant movement popular in the mid‐nineteenth century (Parramore et al. 2000; Fogarty 2001). In December 1856, a fire in a neighboring building spread to Saint Patrick's, destroying the church (Fogarty 2001). It was one of several churches burned in the city (Fogarty 2001), with the fires rumored to have been set by the Know‐Nothings (Pollard 2019). In response, Father O'Keefe, a priest at Saint Patrick's, embarked on a massive fundraising campaign to rebuild the church and maintain the Catholic presence in Norfolk. He was able to raise funds for the construction of another church on the grounds of Saint Patrick's cemetery—Saint Mary's—which was dedicated in October 1858 (Fogarty 2001; Rose 2007; Pollard 2019).

In the 1860s, the historically Black Catholic congregation of **Saint Joseph's** was established in Norfolk (Parramore et al. 2000). Over its first 25 years of existence, the parish became a cornerstone of the Black Catholic community, baptizing more than 800 people and converting over 500 persons (Pollard 2019). In 1889, Saint Joseph's founded **Saint Joseph's Colored School**, one of the first Catholic schools in the city (Rose 2007). The parish remained a vital institution for the community until the 1960s, when it was demolished as part of urban renewal projects. Following its closure, the Black Catholic congregation was consolidated with that of **Saint Mary's**, which became the primary church serving Norfolk's Catholic population (Rose 2007).

**FIGURE 1:**
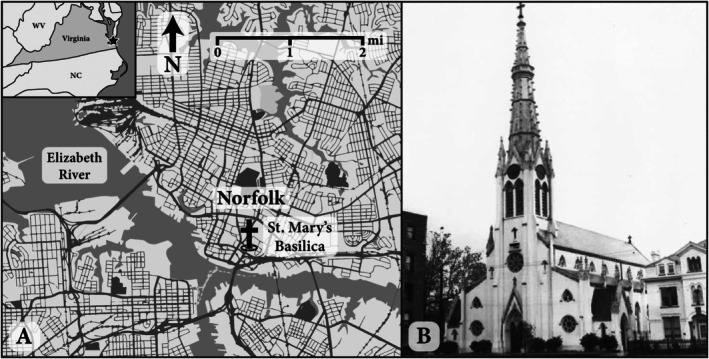
(A) Map of Norfolk, Virginia (USA) with the Basilica of Saint Mary's of the Immaculate Conception labeled. (B) Image of Saint Mary's church taken in 1953. Image reproduced from Yarsinske (1999).

Saint Mary's gained recognition as a historic site in 1979 when it was listed on the **National Register of Historic Places** for its significance in the history of Catholicism in Norfolk (Pollard 2019). The church was subsequently designated a basilica in December 1991, formally becoming the Basilica of Saint Mary's of the Immaculate Conception (Figure 1) (Fogarty 2001). In 2019, its nomination was updated to reflect its **basilica status** and to highlight additional details about its architectural significance (Pollard 2019). As part of this process, renovations were undertaken, ultimately contributing to the development of the project outlined below.

## 2 Project Background and Community Engagement

### 2.1 Ethical Orientation

Human skeletal remains can be understood as the physical representation of past living individuals, embodying the personhood of the deceased individual themselves, as well as the cultural and spiritual beliefs of the living communities who connect with them (Lambert and Walker 2018). Research on archaeological individuals (human remains) therefore requires a deliberate ethical orientation to consider the potential impacts on both the decedents and any connected living communities (Fleskes et al. 2022; Agarwal et al. 2024). As a part of this process, it is critical to identify and engage connected communities to obtain proxy consent, or permission, to carry out bioarchaeological research (Gibbon et al. 2024; Wagner et al. 2020).

For this project, we employed a community‐based research framework to create an ethically informed and community sensitive collaborative effort that centered the needs and questions of connected communities. This approach has been successfully implemented in other archaeological projects to ensure that the research methods and resulting products minimize harm and maximize benefits for the affected communities (Atalay 2012; Blakey 2009; Gilmore et al. 2024).

For this project, the communities that were identified and engaged to provide proxy consent included the contemporary congregation of Saint Mary's Basilica and the Virginia Department of Historic Resources. We also consulted members of the surrounding community and other persons connected to the basilica. In what follows, we detail how we utilized a community‐based engagement model to work collaboratively with our identified proxy community.

### 2.2 Project Development and Community Engagement

In 2016, Saint Mary's began significant renovations to restore the historic church, including structural repairs and interior upgrades. Recognizing the likelihood of burials beneath the church, several of which are visible directly outside of the building to the north, the restoration consultants Commonwealth Preservation Group (CPG) recommended that the church leadership enlist DATA Investigations to survey the church grounds. This organization is a cultural resources firm of the Fairfield Foundation, a non‐profit organization dedicated to involving the public in hands‐on archaeology, preservation, and education activities within Norfolk and surrounding areas. DATA Investigations filed an anticipatory burial permit with the Virginia Department of Historic Resources (DHR) and provided on‐site monitoring during any ground disturbances. Through their mapping work, DATA Investigations found evidence of at least 27 burials inside and immediately adjacent to the basilica (Brown et al. 2025).

Direct engagement with the congregation, involving invested community members and institutional stakeholders through in‐person tours and virtual meetings, led to growing interest in conducting archaeogenomic research. A series of in‐person and virtual community forums were held to discuss whether the persons buried beneath Saint Mary's church (hereafter referred to as “Congregants”) should be disinterred and studied. Archaeologists from DATA Investigations detailed the advantages and limitations of analyzing human remains through osteological and genetic methods and described the potential invasiveness and/or destructiveness of these techniques. After these deliberations, the consulted community collectively chose to pursue a focused scientific study of the earlier congregation and agreed that the disinterred remains would be reburied in the nearby Saint Mary's Catholic Cemetery following excavation and analysis.

The Virginia DHR approved the church's burial recovery permit (#2019‐0667) in 2020. Following the renovation schedule for the basilica, burial recovery was scheduled for May 2020, which coincided with the first wave of the COVID‐19 pandemic in the United States. As a result, strict distancing measures were implemented, including mandatory mask wearing and a limit of only 10 people at the site during excavations. Despite these restrictions, DATA staff facilitated limited visits for vested congregation and community members with appropriate COVID precautions to ensure transparency throughout the excavation.

Following excavation and osteological analysis of the recovered burials, the research team presented the preliminary archaeological and osteological findings to the Saint Mary's congregation. This presentation was conducted at the basilica in 2022 after large gatherings were deemed safe in the post‐pandemic period, as well as broadcast via Facebook Live and the congregation's Zoom channel to accommodate ongoing public health considerations. The presentation described the major takeaways from the archaeological research as well as the preliminary identifications of genomic ancestry (or population affinity), sex, and lived experience for the Congregants studied.

Importantly, this meeting also offered the opportunity to discuss potential future aDNA analyses with church members. This discussion was integral to the informed consent processes, following other community engagement models for aDNA research (Fleskes et al. 2021), to ensure that true informed consent could be granted by the proxy community. The meeting presentation therefore also included an overview of DNA analysis with a focus on the laboratory methodologies used for aDNA extraction. As a part of this effort, attendees were shown a 3D virtual tour of the aDNA laboratory at the University of Connecticut to assist them in visualizing laboratory spaces and processes, in line with other approaches using aDNA visualization techniques for community engagement purposes (Fleskes et al. 2025). We also discussed the questions that can be answered using aDNA, as well as the limitations of this research approach.

The presentation concluded with a list of ethical commitments by anthropological geneticists Fleskes and Schurr (Table 1). Following the presentation, we took time to answer audience questions. We sought feedback concerning future publication and data management strategies, including whether any genomic data generated from the Congregants should be made open access or remain protected under restricted data‐use agreements.

**TABLE 1:** List of ethical commitments related to the ancient DNA research. Part of the Ancient DNA & The Basilica of Saint Mary's of the Immaculate Conception's community research presentation given on September 09, 2022.

List of Ethical Commitments Related to the Ancient DNA Research: “Promises from Us”
1. DNA will only be used for purposes approved by the Community.
2. The DNA samples will only remain in custody of designated persons for the allotted period to conduct DNA testing.
3. All DNA results will be reported to the Community first.
4. We commit to making the science as transparent as possible.

As a result of this meeting, the congregation approved destructive aDNA analysis of the human remains. A burial recovery permit amendment was subsequently filed and approved by the DHR. Destructive aDNA analyses were conducted between 2022 and 2023. Given continuing pandemic constraints, we shared real‐time updates on the aDNA testing, including written letters and video summaries, through virtual channels and social media platforms, including the Facebook and Twitter pages of Saint Mary's Basilica and the Fairfield Foundation.

We presented the final genetic results detailed in this manuscript during an in‐person community meeting at the basilica in November 2024. This presentation was also streamed on Facebook Live through the basilica's Facebook page to accommodate those who could not attend in person. During this presentation, we provided a short summary of the archaeological and osteological analyses completed to date, followed by a description of the aDNA findings and their implications for understanding the church community's history. We also discussed genomic data management risks and benefits during the question‐and‐answer period. Those persons in attendance did not feel strongly about limiting data access, which we later confirmed through personal discussions with other congregation members. As a result, the data generated in this study are publicly available (see Data Availability Statement).

## 3 Archaeological Findings

Archaeological excavations and burial recovery took place in March 2020. The burials selected for recovery were chosen based on consultation with the congregation, as well as recommendations from DATA Investigations regarding excavation feasibility, the likelihood that the skeletal remains would be well preserved, and variation in interment style. The portion of the Saint Patrick's cemetery exposed beneath the floor of Saint Mary's Basilica indicated that the burial ground was probably at or near full capacity. Additional remote sensing conducted *pro bono* by David Givens and Jamestown Rediscovery confirmed a densely packed cemetery that contained hundreds of burials extending beneath much of the current sanctuary and probably beyond the altar to the east.

**FIGURE 2:**
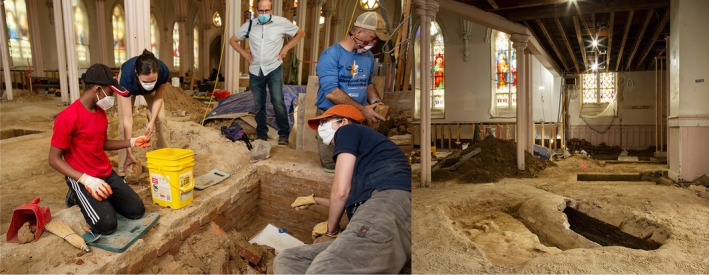
Photographs of the excavation of the burials at the Basilica of Saint Mary's of the Immaculate Conception. Photos used with permission of Brian Palmer (brianpalmer.photos). Left image shows the team excavating Crypt 1, including Keenan Javon Hurdle, Raquel Fleskes, Michael Clem, David Brown, and Joanna Wilson Green (left to right). The image on the right depicts the excavation area in the narthex of the basilica, showing Burial 7 next to the exposed drainage tunnel, with Crypt 3 and Crypt 2 behind.

**FIGURE 3:**
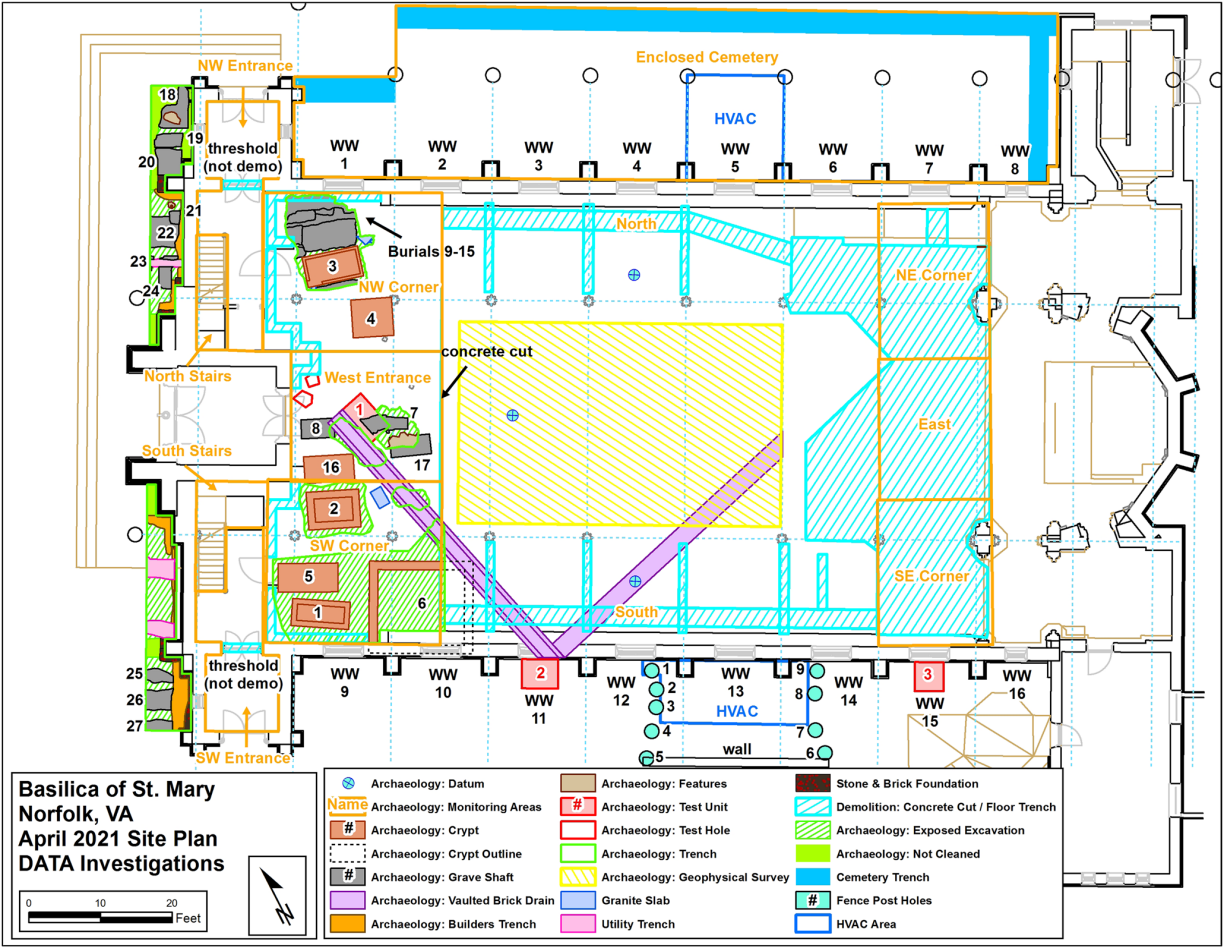
Archaeological site map for Saint Mary's basilica produced by DATA Investigations. The entrance to the basilica is located on the left‐hand side of the diagram between the North and South Stairs, with the inner Sanctuary found in the furthest right‐hand area of the map. Burials that were excavated in this study are found near the entrance of the basilica at the narthex and are numbered 1, 2, 3, 7, and 8.

Based on this evidence, Crypts 1–3 and Burials 7 and 8 were excavated (Table 2; Figures 2 and 3). Burials were generally oriented in the expected east–west direction, although aligning more closely with the city grid rather than the cardinal directions. Burial types included handmade brick vaulted crypts bonded with oyster shell mortar (Crypt 3), machine‐made brick and sand mortar “lawn crypts” that accommodated multiple individuals (Crypt 1 and 2), and simple grave shafts cut deep into the soil (Burials 7 and 8). Brick crypts were further assigned to two broad periods in the cemetery's history, with handmade brick crypts likely used earlier (1790s to 1820s) and machine‐made brick crypts used during a later period (1830s to 1858).

**TABLE 2:** Archaeological findings for the burials at Saint Mary's Basilica.

Burial number	Burial description	Material culture	Estimated date range (AD)
Crypt 1	Machine‐made brick and sand mortar vault; Could accommodate multiple individuals; Wood coffin	Hinges, Handles, Nails, Bone buttons	1830s to 1858
Crypt 2	Machine‐made brick and sand mortar vault; Could accommodate multiple individuals	Coffin fragments, Iron support beam	1830s to 1858
Crypt 3	Handmade brick vaulted crypt; Bonded with oystershell mortar; Wood coffin	Nail fragments	1790s to 1820s
Burial 7 (7A)	Grave shaft with wood coffin; Covered by thick layer of crushed mortar or lime; Buried with 7C	Pins, Bone buttons	1790s to 1858
Burial 7 (7C)	Grave shaft with wood coffin; Covered by thick layer of crushed mortar or lime; Buried with 7A	Pins, Bone buttons	1790s to 1858
Burial 8	Grave shaft with wood coffin	None	1790s to 1858

Burials without a brick crypt dated to either period and likely contained persons who could not afford more elaborate and expensive interments. It is also possible that burials without brick crypts reflected a need to bury the dead expeditiously. In this regard, it is notable that Burial 7 was covered by a relatively thick layer of crushed mortar or potentially lime, a feature that may reflect fears surrounding the yellow fever pandemic and a belief that this material could better protect the living (Schotsmans et al. 2015). Alternatively, the mortar or lime might instead reflect the intrusion of the initial construction debris related to the erection of Saint Mary's church.

Very little material culture (artifacts, tools, or physical objects) was recovered from the burial excavations (Table 2). Crypt 1 contained the most coffin hardware, including hinges, handles, nails, and bone buttons. In contrast, Crypt 3 contained the least material, with only small fragments of heavily encrusted nails recovered. No material culture was recovered from Burial 8. The burials in Crypt 2 were likely removed before Saint Mary's was constructed, leaving behind fragments of the coffin, an iron support beam, and small traces of human remains. The six‐foot depth of Crypt 2 also suggested that this crypt was intended to inter multiple individuals, most likely from the same family.

Burial 7 included two coffins, with a smaller, child‐sized coffin found resting atop an adult‐sized coffin. The skeletal remains of the former were found on top of the latter, likely representing a child and adult who died in quick succession. Both were found with straight pins and a small number of bone buttons. The tightly packed backfilled clay soils surrounding the coffins created an imprint of the wooden boards as they deteriorated, preserving their grain patterns in the clay. Voids remained within both Burials 7 and 8, enabling the full documentation of the coffins' dimensions (Table 2).

Unique among the interments, Burial 8 was significantly impacted by the construction of a brick‐vaulted drainage tunnel that cut through the cemetery during the height of its use in the early nineteenth century. This was initially thought to be connected to the Underground Railroad, to which Norfolk was connected (Newby‐Alexander 2021). However, analyses of the brick manufacture indicated that the tunnel was produced after this period and, therefore, could not have been used for this purpose.

## 4 Osteological Findings

The skeletal remains of the Congregants were moved to the Archaeology and Forensic Science Laboratory at Towson University for osteological examination in July 2021 (Kollmann 2022). Each bone and tooth element was inventoried and examined for completeness, with any element having 32% or less of its full size inventoried as a fragment and not included in the total bone counts (Owsley and Jantz 1989; Langley et al. 2016). Taphonomic changes were assessed following Behrensmeyer (1978) and characterized on a scale of one to five, with one representing the best preservation and five being the worst. Osteological age, sex, ancestry, and pathology were estimated following established protocols (Gill and Rhine 1990; Buikstra and Ubelaker 1994; Bass 2005; Komar and Buikstra 2008; Burns 2015).

Of the five interments excavated, four contained human remains representing a total of at least five individuals (Table 3). The preservation of these remains ranged from moderate to poor, with no fully complete skeletal or dental assemblages being present. Most bones exhibited an orangey‐brown hue. However, some remains had a darker coloration due to coffin wood staining, while others exhibited a pinkish tint from brick staining. Copper staining, likely from a straight pin, was also observed on one skeletal element from Crypt 3, suggesting this individual was shrouded (Pokines and Baker 2021).

**TABLE 3:** Osteological findings from burials at St. Mary's Basilica. MNI (miniumum number of individuals), as well as estimations of age, sex, and ancestry presented in this table are based on osteological assessments. Taphonomic preservation is based on Behrensmeyer (1978). Original data is reported in Kollmann (2022).

Burial number	MNI	Preservation	Completeness	Sex	Age	Ancestry	Dental pathology	Skeletal pathology
Crypt 1	1	Moderate preservation (Behrensmeyer code 3)	Incomplete	Male	35–49 years	African ancestry due to femoral diaphysis	Dental caries; Moderate occlusal wear; Dental calculus present	Slight arthritic lipping and right femoral condyle, right patella and right humerus; Healed periostitis on distal right fibula; Coffin wood staining
Crypt 2	0	N/A	N/A	N/A	N/A	N/A	N/A	N/A
Crypt 3	1	Poor Preservation (Behrensmeyer code 4)	Very Incomplete	Possible Female	Older Adult	Crenulated occlusal surface of maxillary third molar suggestive of African ancestry	Dental carie; Heavy occlusal wear	N/A
Burial 7 (7A)	2	Moderate preservation (Behrensmeyer code 3)	Incomplete	Probable Female	25–39 years	Indeterminate	Antemortem tooth loss; Dental carie; Moderate wear; Dental calculus present	Smorl's depressions on three thoracic vertebrae; Slight lipping in left talus
Burial 7 (7C)	2	Poor Preservation (Behrensmeyer code 4)	Incomplete	Indeterminate	Infant (3–6 months)	Indeterminate	Hypoplastic bands on maxillary incisors	N/A
Burial 8	2	Poor Preservation (Behrensmeyer code 4)	Incomplete	Possible Female	Older Adult	Indeterminate	Dental caries present; Active abscess on right I^1^; Enamel hypoplasia on right I^2^	Duplication of right innominate with acetabulum only; Widespread joint surface pathology observed, including arthritic lipping, porosity and eburnation; Healed fracture to metacarpal; Smorl's depressions on vertebrae; Coffin wood staining

Most of the burials (Crypts 1 and 3) contained the remains of one individual, with Burials 7 and 8 having at least two individuals. As noted above, Burial 7 included an infant (Burial 7A; aged 3–5 months) lying above a probable young adult female (Burial 7C; aged 25–39 years). Burial 8 contained the remains of an older adult female and a duplicate right innominate (right half of the pelvis bone), suggesting the previous presence of another individual, although no other duplicate skeletal elements were identified. This situation most likely occurred due to the structural impact of the vaulted brick drainage tunnel construction that cut into the burial and subsequent indiscriminate movement of human remains. Thus, while the assessment of the minimum number of individuals for Burial 8 is two, a single characterization of age, sex, and ancestry was given as an adult female.

Due to the poor preservation and incomplete nature of the skeletal remains, osteological assessments of age, sex, ancestry, and pathology were limited but offered a preliminary glimpse into the demographics of the Congregants buried at Saint Patrick's (Table 3). As noted above, four of the Congregants were adults, and one was an infant (Burial 7A). Of the adults, three were estimated to be probable or possible female sex, one estimated to be male, and another judged indeterminate due to its poor preservation.

An examination of dental health offered additional insights into the decedents' life experiences. Dental pathology consisted of caries, antemortem tooth loss, and moderate wear. Of the teeth present in the assemblage, 13 of them (17%) showed carious lesions, and 10 elements (15%) showed antemortem tooth loss as evidenced by alveolar reabsorption in the mandible, or regeneration of bone due to tooth loss that is often due to periodontal disease (Mays 2014). In addition, evidence of stress during the life course was seen in the presence of enamel hypoplasias, or defects in tooth enamel typically resulting from physiological stress during childhood development, on the maxillary incisors of the Congregant in Burial 8 (Figure S1) as well as the infant in Burial 7C. The Congregant in Burial 8 further displayed an active abscess, although it was unclear if it contributed to the cause of death. Overall, the cause and manner of death could not be determined based on the present skeletal assemblage.

Skeletal pathology was likely underreported due to poor preservation but nevertheless informed our understanding of the life history of the interred individuals. For example, a healed fracture of a metacarpal was seen on the remains from Burial 8 (Figure S2), as well as a healed depression fracture on the cranium of the Congregant in Crypt 1 (Figure S3), suggestive of past injuries. Also notable for the Crypt 1 Congregant was evidence of healed periostitis on the right distal fibula likely related to an ankle injury (Figure S4). Most bone pathologies seen in the assemblage were related to age degenerative processes, as shown through erosion, porosity, and lipping at joint surfaces (Figures S5–S7). Schmorl's depressions in the vertebrae of the Congregants in Burial 7A and Burial 8 (Figure S8) are associated with heavy labor. Overall, the osteological examination of the skeletal remains suggested that the Congregants, especially those in Crypt 1 and Burial 8, experienced non‐life‐threatening injuries and age‐related pathology over the course of their lifetimes.

## 5 Ancient DNA Findings

### 5.1 Lab Analyses

Skeletal elements from five Congregants (Crypts 1 and 3, Burials 7A, 7C, and 8), including petrous‐temporals, a metatarsal and phalange, were selected for genetic analysis based on their preservation at the time of burial recovery (Table 4; Table S1). These samples were transported to the University of Connecticut's aDNA lab for analysis. This lab is a restricted access BSL‐1, ISO‐6/7 certified facility designed to minimize exogenous DNA contamination through the use of overhead UV lights, positive pressured unidirectional air flow, and mandatory personal protective equipment, as well as separate sample preparation, extraction, and library preparation rooms.

**TABLE 4:** Ancient DNA sequencing, mapping, and uniparental haplogroup results for Congregants buried at St. Mary's Basilica. Percent mtDNA authenticity was generated using ContamMix (Fu et al. 2013).

Burial number	Testing ID	Bone element tested	Whole genome coverage	% Reference coverage > 1×	Percent endogenous DNA	Genetic sex estimation	Y‐Chromosome haplogroup	MtDNA coverage	MtDNA haplogroup	% MtDNA authenticity
Crypt 1	STM1	Left Petrous	0.10×	0.04	1.86%	XY	R‐Y128530	54.63×	H1b1 + 16,362	95%
Crypt 3	STM2	Metatarsal	N/A	N/A	N/A	N/A	N/A	N/A	N/A	N/A
Crypt 3	STM3	Right Petrous	0.37×	0.15	6.65%	XY	R‐FT8333	112.28×	U5a1b1a2	97%
Burial 7 (7A)	STM7A	Petrous Fragment	0.001×	0.001	0.02%	N/A	N/A	7.82×	H2a2a	N/A
Burial 7 (7C)	STM7C	Petrous Fragment	0.002×	0.001	0.04%	N/A	N/A	6.13×	R0	86%
Burial 8	STM8	Phalange	0.60×	0.25	4.09%	XX	N/A	114.25×	L3f1b	96%

We extracted ancient DNA following the Dabney and Meyer (2019) protocol, and built double‐stranded DNA libraries with a partial UDG digestion (Carøe et al. 2018). Indexed libraries were then enriched for human whole genome DNA (Arbor Biosciences). Five enriched libraries (Crypt 1, Crypt 3, Burials 7A, 7C, and 8) contained high enough concentrations of DNA for sequencing on a MiSeq Nano (2 × 33), followed by HiSeq X (2 × 150) sequencing at Admera Health (New Jersey, USA). All genomic data produced in this project are publicly accessible in the Sequence Read Archives under accession number PRJNA1293775. More details on these methods can be found in Text S1.

### 5.2 Sequence Data Analyses

Sequence adapters were removed using AdapterRemoval (Schubert et al. 2016), and trimmed reads mapped to the human reference genome (hg19) and mitogenome (NC_012920.1). Mapping and coverage values were generated using Qualimap (Okonechnikov et al. 2016). Damage patterns were assessed using MapDamage2.0 (Jónsson et al. 2013). Contamination was characterized by examining the X‐chromosome for genetic males using ANGSD (Moreno‐Mayar et al. 2020), and the mitochondrial DNA (mtDNA) using ContamMix v.1.0‐10 (Fu et al. 2013) and HaploCheck (Weissensteiner et al. 2021).

Pseudo‐haploid variants were called against the Human Origins Reference Panel (Lazaridis et al. 2014), and principal components analysis was conducted using SmartPCA (Price et al. 2006) using the lsqproject option. ADMIXTURE was run (Patterson et al. 2012), with the results visualized in R using pophelper (Francis 2017). Outgroup F‐statistics were calculated using popstats (Skoglund et al. 2015). Genetic sex was estimated by comparing the ratio of sequences mapped to the X‐ and Y‐chromosomes following Skoglund et al. (2013), and genetic kin relationships were examined using READ (Kuhn et al. 2018).

For genetic males, Y‐chromosome haplogroups were determined using Yleaf (Ralf et al. 2018), and for all individuals, mtDNA haplogroups were classified using Haplogrep3.0 (Weissensteiner et al. 2016). Comparative mtDNA analysis was also conducted for Congregant in Burial 8 due to its unique haplotype profile using 111 mitogenome sequences downloaded from Genbank (Pardiñas et al. 2014). These sequences were used to create a maximum likelihood tree using IQ‐tree (Nguyen et al. 2015). The resulting tree was visualized using FigTree (http://tree.bio.ed.ac.uk/software/figtree/). Additional details can be found in Text S1.

### 5.3 Ancient DNA Results

We successfully extracted DNA at varying concentrations (Spreadsheets S2 and S3), yielding genomic coverage between 0.001× and 0.6× (Table 4). Damage plots showed characteristic patterns for partial UDG‐treated aDNA for all samples (Figure S9). Mitochondrial contamination estimates ranged between 3% and 14%, and X‐chromosome contamination was less than 0.5% for genetic males (Table 4; Table S2; Spreadsheet S4). All together, we found that three Congregants (Crypt 1, Crypt 3, and Burial 8) had sufficient genomic coverage (> 0.1×) and low contamination estimates to move forward with downstream autosomal analyses (Table 4).

Analysis of autosomal relatedness was conducted to determine if the three Congregants were biologically related to each other. Given the lack of sufficient genomic coverage, the adult and infant in Burial 7 (Burial 7A and 7C) were not tested. No evidence of relatedness was detected between the Congregants in Crypt 1, Crypt 3, and Burial 8 (Figure S10). Genetic sex estimates suggested that the Congregants in Crypt 1 and Crypt 3 were chromosomal males with Y‐chromosomal DNA, and the Congregant in Burial 8 was a chromosomal female (Table 4; Spreadsheet S3). These results matched osteological estimates of sex for the Congregants in Crypt 1 and Burial 8, but not for the Congregant interred in Crypt 3.

**FIGURE 4:**
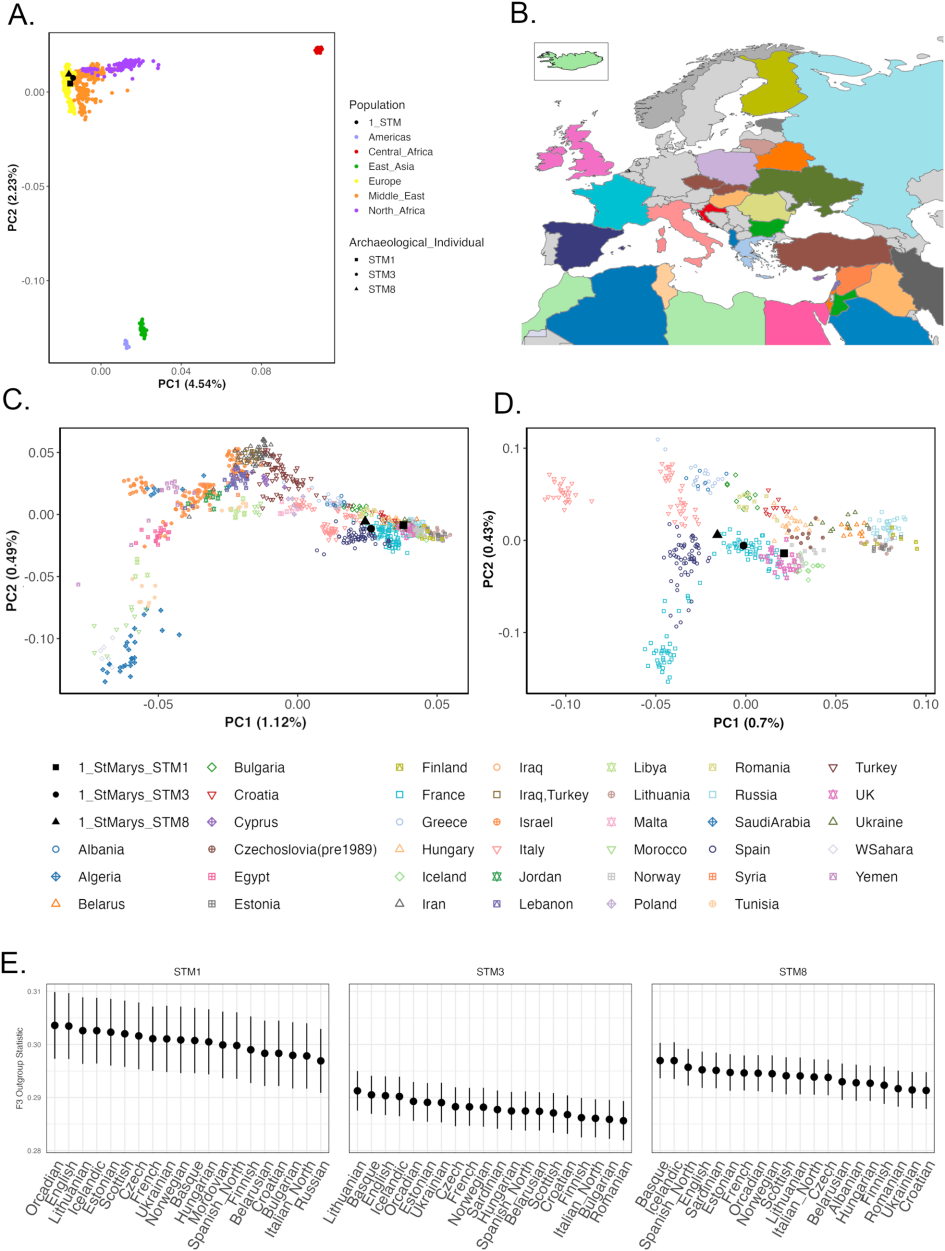
Population affinity characterization using SmartPCA and outgroup F‐Statistics. (A) PCA of worldwide populations, with the Congregants labeled in black. (B) Map showing the geographic locations of comparative European, Middle Eastern, and North African populations used from the Human Origins reference panel used for (C) PCA including only European, Middle Eastern, and North African populations and (D) PCA including only European populations. Symbols for populations in panels C and D are shown below, with colors corresponding to the map in B. European populations include: France, Italy, UK, Russia, Bulgaria, Hungary, Lithuania, Belarus, Ukraine, Estonia, Czechoslovakia, Iceland, Greece, Spain, Finland, Malta, Croatia, Poland, Norway, Albania, Cyprus, and Romania; Middle Eastern populations include: Israel, Iran, Syria, Lebanon, Jordan, Saudi Arabia, Turkey, Iraq; North African populations include: Algeria, Yemen, Egypt, Libya, Tunisia, Western Sahara, and Morocco. Percent variance is noted for each PC dimension on the X and Y axes. (E) Outgroup F‐Statistics for the Congregants in Crypt 1 (STM1; left), Crypt 3 (STM3; middle), and Burial 8 (STM8; right). Top twenty populations are shown.

Biparental ancestry was estimated from autosomal DNA variants using PCA, outgroup F‐statistics, and ADMIXTURE, with comparative populations drawn from the Human Origins reference panel (Spreadsheet S2). Analysis with SmartPCA using global and regional population panels (Figure 4A; Figure S11) suggested that all three Congregants were genetically affiliated with present‐day European or Near Eastern populations. Based on these results, a subsequent PCA using only European, Near Eastern, and North African populations was run; it also revealed that all three Congregants displayed genomic affinity with European populations (Figure 4B,C). Further comparative analysis with only European population data showed an association with present‐day populations living in Spain, the United Kingdom, and France (Figure 4D). Specifically, the Congregant in Crypt 1 was the most genomically similar to contemporary populations in the United Kingdom, whereas those in Crypt 3 and Burial 8 clustered with populations in France and Spain, respectively.

As an additional measure of population affinity, outgroup F‐statistics were run to explore which regional populations shared more genetic drift with the Saint Patrick's Congregants (Spreadsheet S3). All three Congregants showed the most similarity to northern and western European populations (Figure 4E), mirroring the results of the PCA analysis. Notably, the Congregants in Crypt 3 and Burial 8 showed affinities with Basque and northwestern European populations, respectively.

**FIGURE 5:**
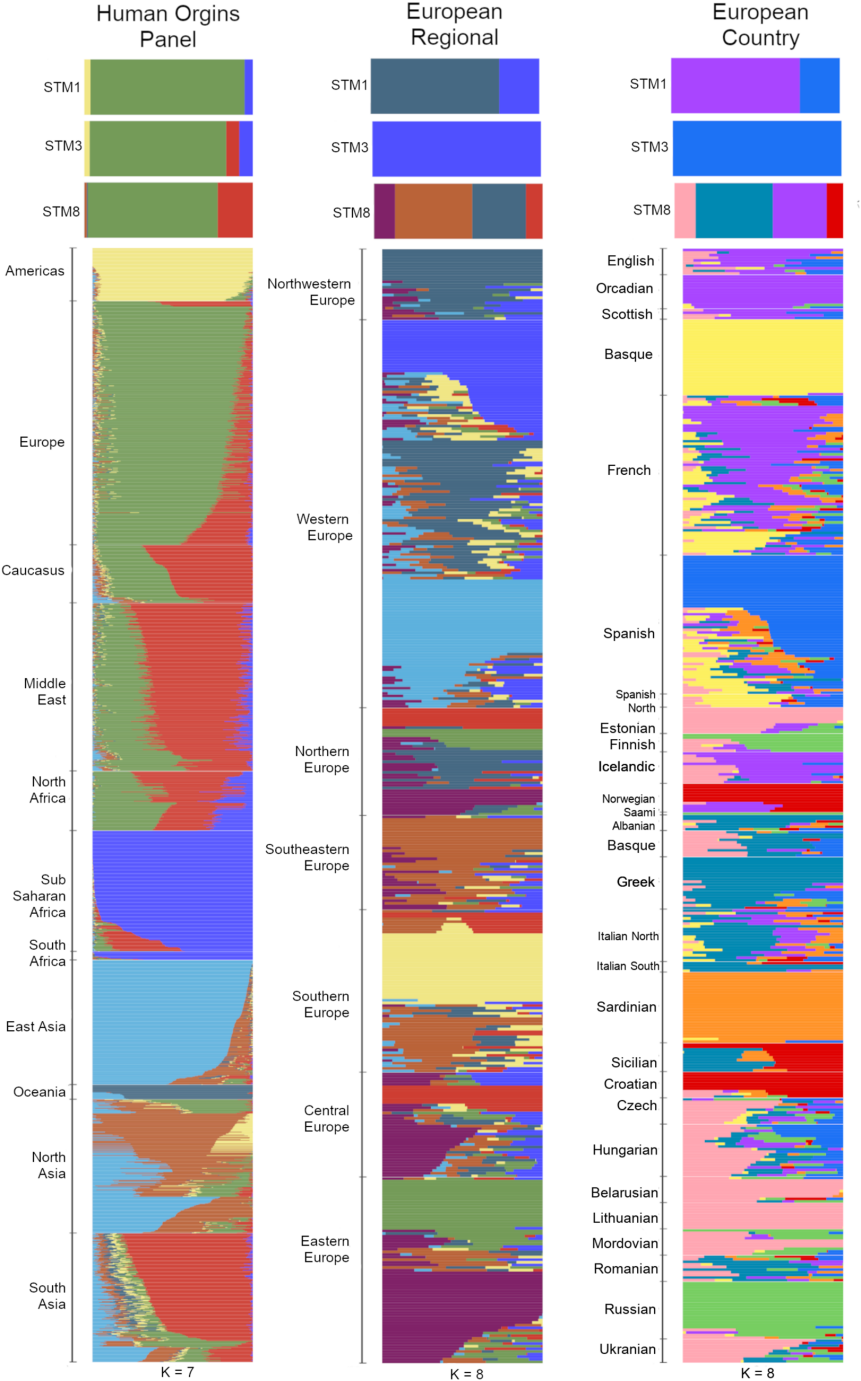
ADMIXTURE analysis for the Congregants in Crypt 1 (STM1), Crypt 3 (STM3), and Burial 8 (STM8), with the left panel showing comparisons with the complete Human Origins reference panel at *K* = 7, and the follwing panels showing comparisons with only the European populations shown at *K* = 8 by region (middle) and country (right).

ADMIXTURE analysis was also undertaken to determine if any Congregants showed evidence of inter‐ and intra‐continental population admixture present. We ran ADMIXTURE analysis for *K* = 5 to 11, again using comparative populations from the Human Origins reference panel (Figure S12), and identified profiles associated with European populations for all three Congregants (Figure 5). To more deeply explore regional European population affiliations, ADMIXTURE was completed using *K* values four through nine with only European comparative populations from the Human Origins panel. Results organized by region (Figure 5; Figure S13) showed northwestern and western European affiliations for Congregants 1 and 3, and less easily distinguishable regional population affinity for the Congregant in Burial 8. When further organized by country (Figure 5; Figure S14), the Crypt 3 Congregant showed clear connections with Spanish populations, and the Crypt 1 Congregant with populations in the United Kingdom. However, the association was less clear for the Congregant in Burial 8.

The Y‐chromosomal haplogroups of the two chromosomal males in Crypts 1 and 3 were analyzed to reconstruct their paternal lineages. Both Congregants belonged to distinct sub‐lineages of haplogroup R1b defined by the M269 and P‐132 SNPs, which represent the most prevalent paternal haplogroup in Europe (Busby et al. 2011; Valverde et al. 2016) (Table 4; Table S3). More specifically, the Crypt 1 male had an R‐Y128530 lineage, classified as haplogroup R1b1a1a2a1a2 using the extended nomenclature system. This lineage also belongs to the R‐U152 branch commonly found in northern Italy, as well as in Switzerland, Poland, Germany, and England (Villaescusa et al. 2017).

Crypt 3 male's lineage R‐FT8333 belonged to haplogroup R1b1a2a1a2a, which is a part of the larger R‐DF27 branch. Interestingly, R‐DF27 is found at high frequencies in Iberia, with ties to the Basque region of northern Spain (Valverde et al. 2016; Villaescusa et al. 2017). This finding supported the autosomal connections with Spanish populations identified through ADMIXTURE analysis (Figure 5), and suggested a significant portion of this Congregant's genomic ancestry came from populations in this region.

Lastly, analysis of mtDNA diversity provided insight into their direct maternal history. The Congregants from Crypt 1 and Crypt 3 had West Eurasian mtDNAs belonging to H1b1 and U5a1b1a2, respectively (Table 4; Spreadsheet S4). H1b is a haplogroup commonly found in central and eastern European populations (Malyarchuk et al. 2018), whereas U5a1 is found more broadly in European populations at low frequencies (Malyarchuk et al. 2010). These haplogroups supported the overall ancestry profile of the Congregants in Crypt 1 and Crypt 3 as being individuals of European descent, with likely connections to contemporary United Kingdom, French, and Spanish populations.

In contrast, the Burial 8 Congregant had a haplogroup L3f1b mtDNA (Table 4; Spreadsheet S4). Associated with African populations, this L3f clade is believed to have originated in Eastern Africa around 50,000 years ago before spreading across the continent, where it now occurs at low frequencies (Soares et al. 2012; Černý et al. 2009). Outside of Africa, haplogroup L3f is rare and only sporadically reported in European populations (Pardiñas et al. 2014). Given that the autosomal profile of the Congregant in Burial 8 was predominantly European with no evidence of large African ancestral contributions, a deeper phylogenetic analysis of their L3f1b haplogroup was conducted. Importantly, their DNA profile presented typical patterns characteristic of ancient DNA and low levels of mtDNA contamination, suggesting that the data reported were authentic and relatively free of modern contaminants.

**FIGURE 6:**
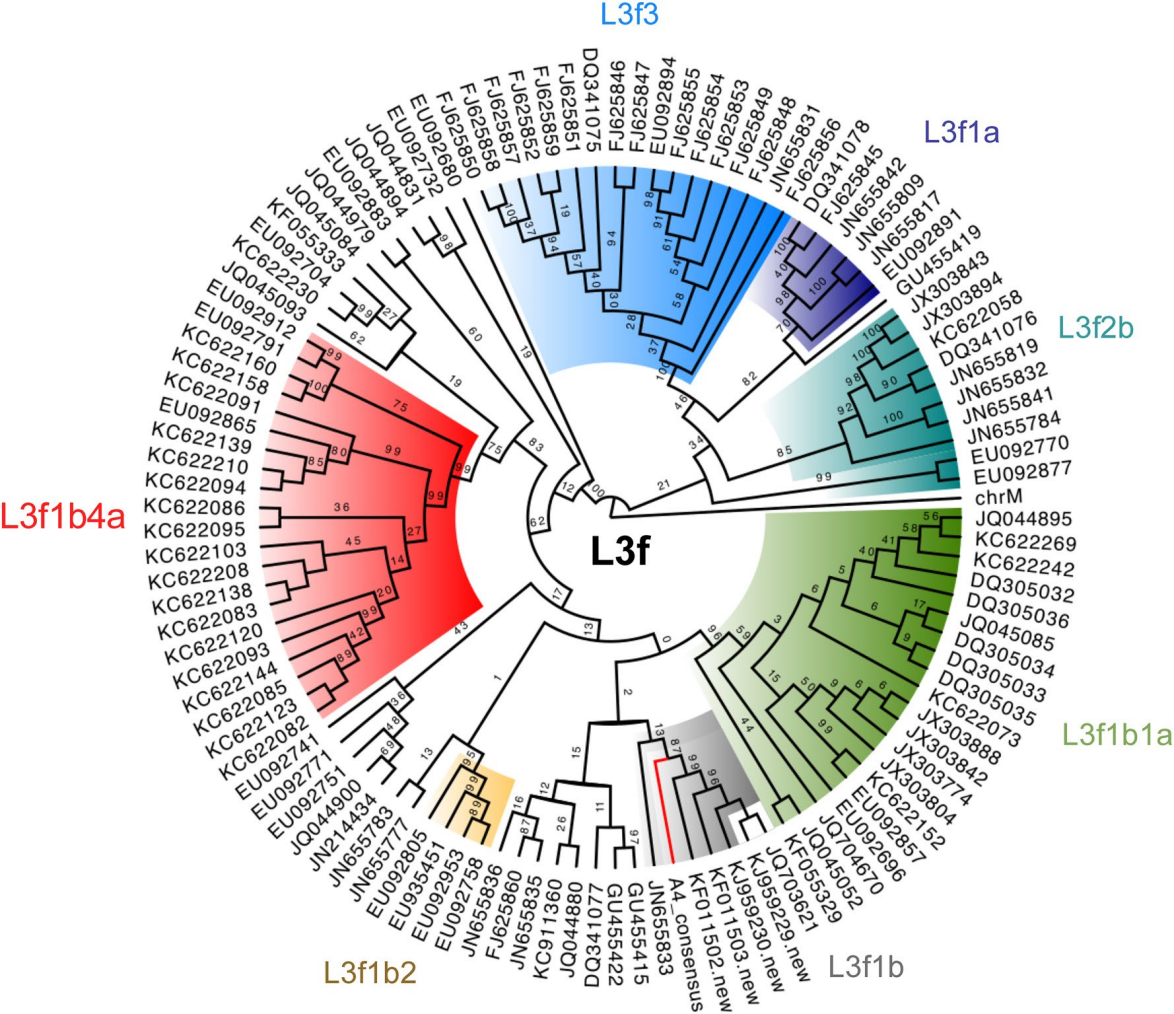
Phylogenetic tree for mitochondrial haplogroup L3f generated using IQ‐tree from comparative reference sequences designated in Pardiñas et al. (2014). The percent bootstrap support is shown for each branch divergence point. Large haplotype subclades are colored for interpretative assistance. The L3f1b haplotype for Burial 8 is shown as a red line within the gray L3f1b subclade, which is supported with 87% bootstrap confidence. Genbank IDs are given for each sample with the exception of the Congregant in Burial 8, which is labeled as A4_consensus.

Reduced median network analysis of comparative L3f mitogenome sequences was conducted using IQ‐Tree (Nguyen et al. 2015; Pardiñas et al. 2014) (Figure 6). The results indicated that Burial 8 Congregant's haplotype was most closely associated with a specific branch of L3f1b found in the Asturias region of northern Spain (Pardiñas et al. 2014). Populations in Asturias have been historically isolated in the Iberian Peninsula (Pardiñas et al. 2012), which is thought to explain the high frequency of this haplogroup there.

Phylogenetic analysis of this Iberian haplotype suggests its parental lineage evolved around 8000 years ago in northern Africa, with deeper roots in West Africa (Pardiñas et al. 2014; Černý et al. 2009). Persons carrying the L3f1b haplotype likely migrated into Spain through the Straits of Gibraltar or across the Mediterranean Sea, hypothesized by others to have occurred through the Moorish expansion in the 12th century AD (Pardiñas et al. 2014). The Spanish maternal connections of the Congregant in Burial 8 are also supported by their autosomal data, which showed genetic affinity with contemporary populations in Spain (Figure 4D). The Burial 8 Congregant is the first archaeological individual reported to carry an L3f1b haplotype, highlighting how migration from Europe has influenced genetic diversity in North America.

## 6 Discussion

The results from our archaeogenomic investigation reveal a complex series of interments that span the history of Saint Patrick's church. We interpret these findings through an intersectional theoretical framework to better conceptualize migration histories and lived experiences within the broader landscape of Catholic Norfolk. Intersectionality, as discussed by Black Feminist theorist Kimberlé Crenshaw, highlights how systems of power differentially shape aspects of individual and/or group identity, such as race, gender, and sex (Crenshaw 1989). These group identifiers also co‐affect each other, producing complex and historically contingent experiences of identity (Collins 1990; Crenshaw 1991). While the concept of intersectionality is typically evoked in discussions of the living (Cho et al. 2013), we draw from its applications in bioarchaeological theory to explore how perceived aspects of identity—such as population affinity, race, class, sex, and religion—are informed by and are embedded within historical and contemporary structures of power (DeWitte and Yaussy 2020; Torres‐Rouff and Knudson 2017; Prince et al. 2022).

Our use of a community‐engaged framework that is grounded in ethical approaches to human skeletal and ancient DNA research, further builds a contemporary intersectionality that influenced how we conducted this work and interpreted its findings. In carrying out this work, we understood that the Congregants whom we studied for this project were once living persons and not simply objects of study (Tarlow 2006). They lived, breathed, and interacted within the historical context that we are attempting to reconstruct. In doing so, we also acknowledge that, as researchers, we carry our own intersections of power and privilege (Flewellen et al. 2021).

Drawing from Standpoint theory, as conceptualized by Sandra Harding (1991), we further understand that our knowledge is socially situated and inherently shaped by our perspectives and the methodologies that we use to study the past (Atalay 2006; Hodder 1999). We recognize that we cannot fully reconstruct the lived experiences of the individuals at the center of this study. However, by employing a multi‐method approach within an intersectional framework, we are able to present a more nuanced interpretation of the past—one attuned to both historical complexity and the present‐day systems of power in which contemporary descendants and community members are embedded (Torres‐Rouff and Knudson 2017).

We further acknowledge that this project includes genomic data from only three individuals. In this context, we are informed by a growing body of work that emphasizes the value of multi‐method and theoretically engaged approaches to interpreting individual lives within their broader social and historical contexts of the past (e.g., Fricke et al. 2021; Stodder and Palkovich 2012; Mant and Holland 2019; Boutin 2011). Such frameworks illuminate how small sample sizes—often viewed as a limitation—can instead serve as powerful entry points for integrating diverse perspectives into, and sometimes against, hegemonic narratives of the past (Martin et al. 2013). Nevertheless, we do not claim that this sample is representative or statistically significant in a conventional sense. Rather, we situate our work within a bioarchaeological space increasingly attentive to the ethical, interpretive, and community‐engaged possibilities of working with fragmentary yet meaningful data (Robb et al. 2019; Hosek and Robb 2019; Geller 2021).

### 6.1 Ancestry, Migration, and Religion

Tracing the ancestral backgrounds of the Congregants buried at Saint Patrick's provides insight into lives shaped by religious and migrational identities. For all of these individuals, their presence in the churchyard of Saint Patrick's suggests that they belonged to the congregation or practiced Catholicism, although the extent of their participation in the Catholic community cannot be directly ascertained. Research on Norfolk's Catholic community at Saint Patrick's (1790s–1856) indicates a predominantly immigrant congregation, with many priests originating from France or Ireland and living in a historically English society (Fogarty 2001). Autosomal DNA evidence largely supports this finding, with all Congregants analyzed in this study being genetically connected to contemporary reference populations from Europe. These results reflect Virginia's history as an English settlement, with some Catholic populations migrating to eastern North America in search of religious freedom (Fogarty 2001; Parramore et al. 2000).

However, Catholic migration to Norfolk was neither uniform nor linear. Rather than representing a single movement, migration histories reflect a complex, multilayered process shaped by diverse motivations (Tsuda 2011). For example, the specific migratory path of the Congregant interred in Crypt 1 is unclear; whether he arrived directly from England or instead lived in other colonial regions before his burial at Saint Patrick's cannot be known without additional isotopic testing. As a European male, he likely had greater mobility within the historical landscape. Likewise, the factors influencing his migration and settlement in Norfolk may have included economic opportunities, religious affiliation, and/or personal circumstances; though definitive reasons will remain uncertain without direct documentation.

Uniparental DNA lineages offer additional evidence of varied migration histories and complex ancestries. The Congregant in Crypt 3 had a Y‐chromosome from haplogroup R‐D27 that appears at high frequencies in the Basque region of northern Spain (Villaescusa et al. 2017), suggesting his paternal family originated from this region. Similarly, the mtDNA of the Congregant in Burial 8 belonged to haplogroup L3f1a, a lineage found in populations from northern Spain (Pardiñas et al. 2014). For both Congregants, Spanish or Basque autosomal population affinity was inferred through outgroup F‐statistics, ADMIXTURE, and PCA analyses.

The presence of one Congregant with a mitochondrial lineage found in northern Spain and another individual with a Y‐chromosomal haplogroup concentrated in the Basque region of Spain was unexpected, as Virginia during the 17th and 18th centuries was primarily an English colony and Spanish colonial activities were largely focused in Florida, the American Southwest, and the Caribbean (Fogarty 2001; Parramore et al. 2000). Yet, these findings highlight the interconnected nature of European interactions and historic landscapes. The Atlantic world was linked by maritime trade routes and, as a major port city, Norfolk frequently welcomed ships from various countries, including Spain (Parramore et al. 2000). In addition, Norfolk's port also witnessed thousands of refugees resettling from Saint‐Domingue (Haiti) following the Haitian Revolution (Bell 2020). While Saint‐Domingue was historically a French colony, the island of Hispaniola was originally colonized by the Spanish, who then occupied the eastern part of the island, Santo Domingo (Dominican Republic) (Ponce‐ Vázquez 2014). Alternatively, the Spanish origin of these Congregants could reflect a more deeply intertwined history of Catholicism in Europe prior to colonial immigration (Greer and Mills 2017). Finally, we cannot rule out direct individual or family migration from Spain or a Spanish colony to Norfolk, given the many reasons people moved and resettled in the Americas.

Overall, an intersectional approach challenges us to think beyond ancestral identification as a linear, unidirectional process of movement. Rather, by grounding archaeogenomic evidence within Norfolk's socio‐historical context, we texturize these ancestral connections to think through the various means and ways that people moved (Cabana and Clark 2011). Their reasons are inherently shaped by the historic landscapes they inhabited, as well as their gendered and racialized bodies, which further impacted how they navigated the world. For archaeogenomic research on past populations, weaving these different perspectives together provides a deeper understanding of migration history and lived experience.

### 6.2 Status: Race, Class, and Sex

The finding of European genomic ancestry for three Congregants buried at Saint Patrick's contrasts with the largely African American demographic of the contemporary Saint Mary's community, illuminating the impact of the congregational merger with Saint Joseph's church in the late 1960s (Parramore et al. 2000; Pollard 2019). While we know that both white and Black parishioners attended masses at Saint Patrick's (Fogarty 2001), it likely occurred under the auspices of segregation, where Black practitioners may have been relegated to separate sections of the church. In this regard, it is unclear as to whether the graveyard at Saint Patrick's was segregated following these racial codes or integrated based on shared religious experience. The finding of three persons of European descent could suggest racial segregation of the burials at Saint Patrick's, although it could also be equally attributable to chance as they reflect a smaller subset of a much larger unexcavated burial population.

A broader examination of lived experience and the construction of social identity and race in Norfolk can also be explored through the archaeological record of Saint Patrick's church. Privilege can be observed in cemetery spaces through the spatial and material contexts of burials (Pearson 1982). All burials found were connected to the churchyard of Saint Patrick's. Access to such interment space would have required either religious, social, or financial capital, or a combination of all three (Mytum 2004). In addition, three of the burials were interred in brick crypts that span the early and later time periods of the church, which would have required additional financial and material resources to construct (Riordan and Mitchell 2011).

The brick crypts (1 through 3) were found near wooden coffin interments (Burials 7 and 8), which would have been a more economically accessible means of internment during this time (Mytum 2004). The lack of identifiable coffin hardware meant that we were unable to specifically date their construction. However, the presence of brick crypts from both early and later interment periods interspersed with wooden coffins of either time period suggests that access to financial capital was not the sole requirement for burial at Saint Patrick's. While interment in brick crypts may have served as a marker of socioeconomic privilege embedded in the act of mortuary care, persons without these resources were nonetheless also included in the mortuary community of Saint Patrick's.

The demographic differences between the crypts and burials at Saint Patrick's also speak to differences in lived experience in Catholic Virginia. Those interred in the crypts were identified as older adult males of European descent. Skeletal pathology indicated the presence of healed breaks and degenerative wear on the body, meaning that they likely labored in life but lived into old age. We can infer that their identities as European males, as interpreted from the bioarchaeological record, would have afforded them privileges to navigate the nineteenth‐century colonial landscape as possible landowners, business owners, or laborers in the port city of Norfolk.

By contrast, the Congregants interred in wooden coffins were identified as adult females and an infant. The presence of enamel hypoplasias in the infant and the older adult female in Burial 8, along with Schmorl's depressions and widespread arthritic wear in the adults from Burials 7 and 8, points to a history of physical stress and labor. Their interment in wooden coffins, with two on top of each other, also suggests they may have had limited financial resources, or that their burials were carried out in quicker succession. In the nineteenth century, women often had limited economic and social autonomy (Baker 1984), which may have shaped their access to such resources. These findings suggest that those interred in wooden coffins may have experienced different social, economic, and gendered vulnerabilities than their brick‐entombed counterparts.

## 7 Conclusion

Our archaeogenomic investigation of burials at Saint Mary's offers valuable insights into the history of its predecessor, Saint Patrick's, and suggests that religion, race, migration, and sex all shape the mortuary landscape and lived experiences of life in early Catholic Norfolk. The aDNA data presented here provide the first evidence of nineteenth‐century genomic diversity of Catholic burials in North America and indicate that they were connected to contemporary western and northwestern European populations. The connection to Spanish populations through specific mtDNA and Y‐chromosome haplotypes highlights the continued utility of uniparental data in refining genomic ancestry interpretations. These findings also challenge simplified narratives of English colonial dominance, instead pointing to the numerous multi‐national colonial entanglements of diasporic populations shaping 19th‐century Catholic life in Catholic Virginia. Additionally, the differentiation in burial structures, such as brick crypts for adult males and wooden coffins for females and infants, offers insights into the social and demographic composition of the congregation. These results highlight the power of an intersectional archaeogenomic approach informed by community engaged efforts to explore the lives of historic populations, ultimately engaging with biological and socio‐historical narratives to illuminate the lived experiences of peoples in the past.

## Author Contributions

**Raquel E. Fleskes:** conceptualization, formal analysis, data curation, visualization, writing – original draft, methodology, writing – review and editing. **Horvey M. Palacios:** writing – review and editing. **Hannah Budner:** writing – review and editing. **Dana Kollmann:** methodology, writing – review and editing. **Cassandra Newby‐Alexander:** writing – review and editing, investigation. **Reed Harder:** writing – review and editing, validation, methodology. **Deborah A. Bolnick:** supervision, resources, writing – review and editing. **Marcus Pollard:** conceptualization, writing – review and editing, project administration, resources, funding acquisition. **Paige Pollard:** conceptualization, writing – review and editing, project administration, resources, funding acquisition. **Theodore G. Schurr:** conceptualization, writing – original draft, supervision, project administration, writing – review and editing. **David A. Brown:** conceptualization, writing – original draft, writing – review and editing, project administration, supervision, methodology, investigation, funding acquisition, resources.

## Conflicts of Interest

The authors declare no conflicts of interest.

## Supporting information


**Data S1:** Supporting Information.

**Data S2:** Supporting Information.

## Data Availability

The data that support the findings of this study are openly available in SRA at https://www.ncbi.nlm.nih.gov/sra/PRJNA1293775, reference number PRJNA1293775.
