# Supplementary material for: Archaeogenomic Analysis of Nineteenth‐Century Burials at Saint Mary's Basilica: An Intersectional Analysis of Religion, Race, and Migration
Source: Am J Biol Anthropol. 2025 Aug 14;187(4):e70110. doi: 10.1002/ajpa.70110 (PMC12351519; doi:10.1002/ajpa.70110)
Supplement: Supplementary file 1 — Data S1: Supporting Information. [file AJPA-187-e70110-s002.docx]

**Archaeogenomic Analysis of Nineteenth-Century Burials at St. Mary’s Basilica: An Intersectional Analysis of Religion, Race, and Migration**

Raquel E. Fleskes, Horvey M. Palacios, Hannah Budner, Dana Kollmann, Cassandra Newby-Alexander, Reed Harder, Deborah A. Bolnick, Marcus Pollard, Paige Pollard, Theodore G. Schurr, David A. Brown

Table of Contents

[Supplemental Text 1. Ancient DNA Analyses. 3](#_Toc193794148)

[**Sequence Data Analyses** 5](#_Toc193794149)

[**Supplemental Text References Cited** 7](#_Toc193794150)

[Supplemental Figures 9](#_Toc193794151)

[**Supplemental Figure 1.** Enamel hypoplasia buccal surface of the right maxillary first incisor from the Congregant in Burial 8 9](#_Toc193794152)

[**Supplemental Figure 2.** Healed fifth metacarpal fracture on the distal joint surface from the Congregant in Burial 8. 9](#_Toc193794153)

[**Supplemental Figure 3.** Frontal, parietal and occipital bones forming top of the cranium from the Congregant in Crypt 1. 10](#_Toc193794154)

[**Supplemental Figure 4.** Healed periostitis on the distal fibula from the Congregant in Crypt 1. 10](#_Toc193794155)

[**Supplemental Figure 5.** Marginal lipping of the distal femur from the Congregant in Crypt 1. 11](#_Toc193794156)

[**Supplemental Figure 6**. Duplicate right innominate found in Burial 8. Image reproduced from Kollmann (2021). 11](#_Toc193794157)

[**Supplemental Figure 7**. Eburnation of a vertebral facet of an upper rib from the Congregant in Burial 8. Image reproduced from Kollmann (2021). 12](#_Toc193794158)

[**Supplemental Figure 8**. Slight Schmorl’s depressions on the thoracic vertebrae from the Congregant in Burial 8. 12](#_Toc193794159)

[**Supplemental Figure 9.** Plots showing damage patterns for ancient DNA samples 13](#_Toc193794160)

[**Supplemental Figure 11**. SmartPCA of Congregants with the complete Human Origins reference panel 15](#_Toc193794161)

[**Supplemental Figure 12**. ADMIXTURE analysis using the Human Origins reference panel. 16](#_Toc193794162)

[**Supplemental Figure 13**. ADMIXTURE analysis using only European populations from the Human Origins reference panel, organized by region 18](#_Toc193794163)

[**Supplemental Figure 14**. ADMIXTURE analysis using only European populations from the Human Origins reference panel, organized by country 20](#_Toc193794164)

[Supplemental Tables. 21](#_Toc193794165)

[**Supplemental Table 1**. DNA sample selection and preservation notes. 21](#_Toc193794166)

[**Supplemental Table 2**. X-chromosome contamination estimation. 21](#_Toc193794167)

[**Supplemental Table 3**. Genetic sex assignment. 22](#_Toc193794168)

[**Supplemental Table 4**. Y-chromosomal haplogroup assignment. 22](#_Toc193794169)

# **Supplemental Text 1. Ancient DNA Analyses.**

Skeletal elements were collected using sterile masks and gloves, with excavation tools being cleaned with 10% bleach followed by a 70% ethanol rinse before repeated use. Samples were placed in sterile bags and frozen until transport to the University of Connecticut for ancient DNA (aDNA) analysis. Skeletal samples were defrosted at room temperature for 24 hours, and any remaining dirt was removed using sterile dental cleaning tools and dry Kimwipes.

For petrous-temporal elements, the inner petrous portion was exposed by removing the surrounding cortical bone using a Dremel Handheld Rotary Tool with diamond tipped attachments. For metatarsal and phalange elements, the distal portions were sectioned and the first millimeter of trabecular bone was removed.

Prepared samples were wiped for one minute with a 50% bleach solution using a sterile Kimwipe and rinsed twice with molecular-grade water for one minute each. Samples were dried and placed in a UV crosslinker for 10 minutes on each side.

The decontaminated samples were ground using a Roche Mixer Mill 4000 with stainless steel vails and grinding balls in 30-second intervals at maximum frequency. The resulting compacted powdered bone was loosened with a sterilized mortar and pestle, and 0.2 to 0.27 grams of bone were collected for DNA extraction.

DNA was extracted using a Nucleic Acid Large Volume Kit (Roche) with a negative control (Dabney and Meyer 2019). Samples were incubated in an EDTA-based buffer at 56°C for 24 hours, undergoing 360° rotation. Extracted DNA was quantified on the Qubit 4 Fluorometer using a dsDNA High Sensitivity Kit (Invitrogen) (**Supplemental Table 2**).

Double-stranded DNA libraries with partial-UDG treatment were built with an additional negative control (Carøe et al. 2018). Samples with high concentration were diluted in ddH20 to create a target input concentration of 100 ng in 30 μl. The concentration of adapters was adjusted based on the final input concentration (**Supplemental Table 2**). The prepared libraries were then purified using a MinElute column clean up (Qiagen), eluted into 25 μl of EB buffer heated to 65°C.

DNA libraries were quantified using the QuantStudio 3 (Applied Biosystems) using SYBR green (Thermo Scientific) with IS7 and IS8 primers (Carøe et al. 2018). Extraction and library blanks showed proportionally higher CT values compared to the rest of the DNA samples, except for sample STM2 (an additional sample from Crypt 3), which was removed from further analysis. Samples were diluted based on CT value to give comparable concentrations for indexing PCR (**Supplemental Table 2**).

Libraries were indexed by splitting them into four replicates and amplified using the Kapa HiFi +Uracil kit (Roche) with indexing primers (New England Biosciences). Cycle number was determined based on qPCR CT value (**Supplemental Table 2**). Indexed samples were visualized on a 2% agarose gel, run at 120V for one hour. Replicate samples were then pooled and purified using a MinElute spin column (Qiagen) clean up. Final indexed libraries were quantified on the Qubit using a dsDNA Broad Range Assay (Invitrogen) and Agilent 2200 TapeStation with the D1000 ScreenTape (**Supplemental Table 2**).

Indexed libraries were enriched using the myBaits Expert Whole Genome Enrichment Kit v4 (Arbor Biosciences) with a 24-hour incubation. All relevant steps took place at a 60°C hybridization temperature. Samples were amplified using Kapa HiFi HotStart Ready Mix (Roche). Enriched libraries were quantified using the previous methods described (Supplemental Table 2).

## **Sequence Data Analyses**

The raw sequencing data were first characterized using FastQC v.0.12.2 (Andrews). Adapters were removed using AdapterRemoval v.2 (Schubert et al. 2016), designating a minimum read length of 30. The presence of overrepresented sequences and adapters were checked using FastQC v.0.12.2, with additional trimming completed as necessary. Trimmed reads were mapped to the human reference genome (hg19) and mitogenome (NC_012920) using bwa aln, and filtered for unmapped reads, mapping quality (>30), duplicates, and multiple mappings. For each sample, sequences were merged using samtools v1.3 (Li et al. 2009), and filtered again.

The Human Origins Reference Panel (Lazaridis et al. 2014) was used to call pseudo-haploid variants using samtools mpileup, with minimum mapping and sequencing quality scores of 30, followed up with pileupCaller v.1.5.3 (<https://github.com/stschiff/sequenceTools>). Variant calling statistics were generated using the genoStats tool, as part of the sequenceTools package. The Human Origins reference populations were filtered for overlapping variants using PLINK v.1.9 (Purcell et al. 2007), and merged with the St. Mary’s dataset for comparison.

SmartPCA from EIGENSOFT v.8 (<http://www.hsph.harvard.edu/alkes-price/software/>) was run using lsqproject and visualized RStudio v4.3.1 using ggplot2 (RStudio Team, 2020; Wickham, 2016). Variant data was then LD pruned using indep-pairwise 50 10 0.1 in PLINK, and all comparative reference populations were made pseudo-haploid to run in ADMIXTURE v.1.3 with 100 bootstrap replicates (Patterson et al. 2012).

***Uniparental Haplogroup Characterization***

Mitochondrial DNA variants were characterized using sequences mapped to the mitochondrial reference genome with samtools mpileup, with a minimum base and mapping quality of 30 and depth of 5, and called using bcftools v.1.15.1 (Danecek et al. 2021). Haploid designation was used, and variants were further filtered for only SNPs. Haplogroups were classified from sequence data using Haplogrep v.2.4.0.

For STM8 (Burial 8), a mitochondrial consensus sequence was generated using bcftools for phylogenetic analyses. Comparative mitogenome sequences (*n* = 111) representing overlapping haplogroups with STM8 (Burial 8) were downloaded from GenBank. Sequences were aligned using Clustal Omega v.1.2.3 (Sievers and Higgins 2014) through Geneious Prime v.2023.2.1 (https://www.geneious.com) based on sequence similarity. Nexus files were generated and used to create a maximum likelihood tree using IQ-Tree v.1.6.12 (Nguyen et al. 2015), with 1,000 iterations of ultrafast bootstrap using the auto-substitution model.

## **Supplemental Text References Cited**

Andrews, Simon. FastQC: A quality control tool for high throughput sequence data. https://www.bioinformatics.babraham.ac.uk/projects/fastqc/, accessed January 1, 2025.

Carøe, Christian, Shyam Gopalakrishnan, Lasse Vinner, Sarah S.T. Mak, Mikkel Holger S. Sinding, José A. Samaniego, Nathan Wales, Thomas Sicheritz-Pontén, and M. Thomas P. Gilbert. 2018. Single-tube library preparation for degraded DNA. *Methods in Ecology and Evolution* 9(2):410–419.

Dabney, Jesse, and Matthias Meyer. 2019. Extraction of highly degraded DNA from ancient bones and teeth. In *Ancient DNA: Methods and Protocols*, edited by Beth Shapiro, Axel Barlow, Peter D. Heinzman, Michael Hofreiter, Johanna L A Paijmans, and Andre E. R. Soares, pp. 25–29. Humana Press, New York.

Danecek, Petr, James K Bonfield, Jennifer Liddle, John Marshall, Valeriu Ohan, Martin O Pollard, et al. 2021. Twelve years of SAMtools and BCFtools. *GigaScience* 10(2):1–4.

Jónsson, Hakon, Aurelien Ginolhac, Mikkel Schubert, Philip Johnson, and Ludovic Orlando. 2013. MapDamage2.0: Fast approximate Bayesian estimates of ancient DNA damage parameters. *Bioinformatics Applications Note* 29(13):1682–1684.

Lazaridis, Iosif, Nick Patterson, Alissa Mittnik, Gabriel Renaud, Swapan Mallick, Karola Kirsanow, Peter H. Sudmant, et al. 2014. Ancient human genomes suggest three ancestral populations for present-day Europeans. *Nature* 513(7518):409–413.

Li, Heng, Bob Handsaker, Alec Wysoker, Tim Fennell, Jue Ruan, Nils Homer, Gabor Marth, Goncalo Abecasis, et al. 2009. The Sequence Alignment/Map format and SAMtools. *Bioinformatics* 25(16):2078–2079.

Nguyen, Lam Tung, Heiko A. Schmidt, Arndt Von Haeseler, and Bui Quang Minh. 2015. IQ-TREE: A fast and effective stochastic algorithm for estimating maximum-likelihood phylogenies. *Molecular Biology and Evolution* 32(1):268–274.

Patterson, Nick, Priya Moorjani, Yontao Luo, Swapan Mallick, Nadin Rohland, Yiping Zhan, Teri Genschoreck, et al. 2012. Ancient admixture in human history. *Genetics* 192(3):1065–1093.

Purcell, Shaun, Benjamin Neale, Kathe Todd-Brown, Lori Thomas, Manuel A Ferreira, David Bender, Julian Maller, et al. 2007. PLINK: A tool set for whole-genome association and population-based linkage analyses. *American Journal of Human Genetics* 81(3):559–575.

Schubert, Mikkel, Stinus Lindgreen, and Ludovic Orlando. 2016. AdapterRemoval v2: Rapid adapter trimming, identification, and read merging. *BMC Research Notes*:1–7.

Sievers, Fabian, and Desmond G Higgins. 2014. Clustal omega. *Current Protocols in Bioinformatics* 48(1):3–13.

# **Supplemental Figures**


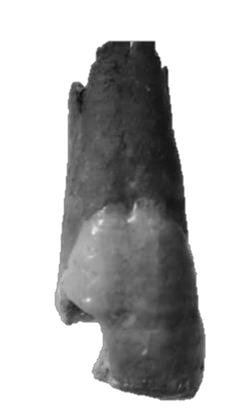


**Supplemental Figure 1.** Enamel hypoplasia buccal surface of the right maxillary first incisor from the Congregant in Burial 8. Image reproduced from Kollmann (2021).


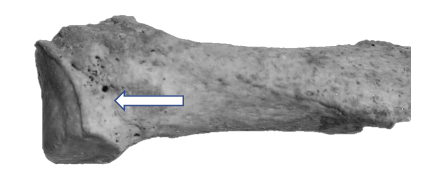


**Supplemental Figure 2.** Healed fifth metacarpal fracture on the distal joint surface from the Congregant in Burial 8. Image reproduced from Kollmann (2021).


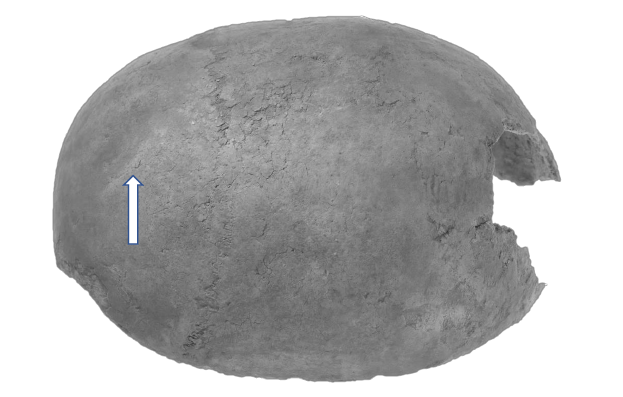


**Supplemental Figure 3.** Frontal, parietal and occipital bones forming top of the cranium from the Congregant in Crypt 1. Arrow shows healed depression fracture. Image reproduced from Kollmann (2021).


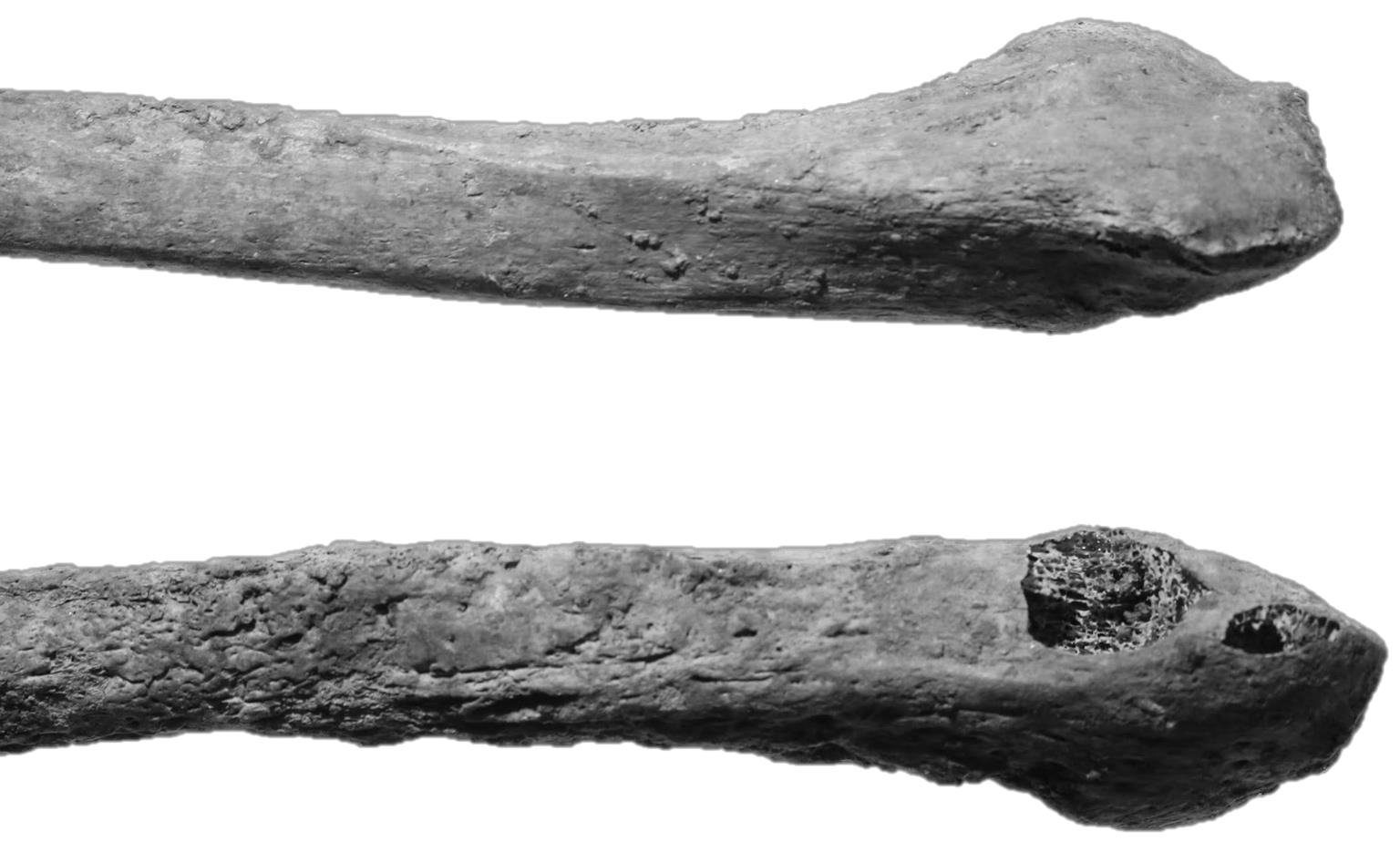


**Supplemental Figure 4.** Healed periostitis on the distal fibula from the Congregant in Crypt 1. Top image is the left unaffected fibula, and the bottom image is the right affected fibula. Image reproduced from Kollmann (2021).


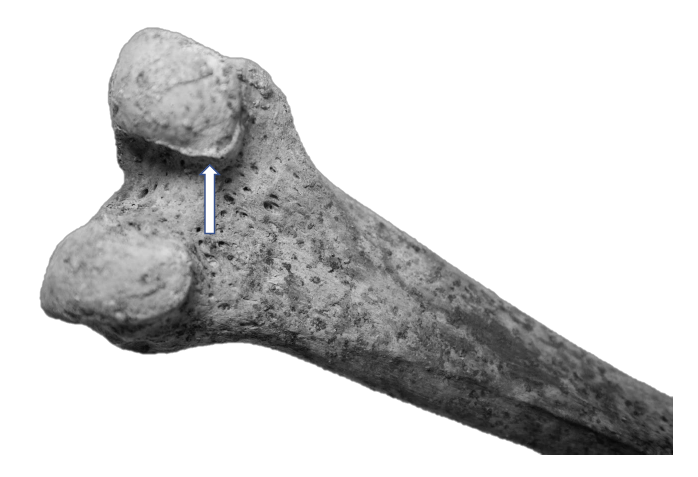


**Supplemental Figure 5.** Marginal lipping of the distal femur from the Congregant in Crypt 1. Image reproduced from Kollmann (2021).


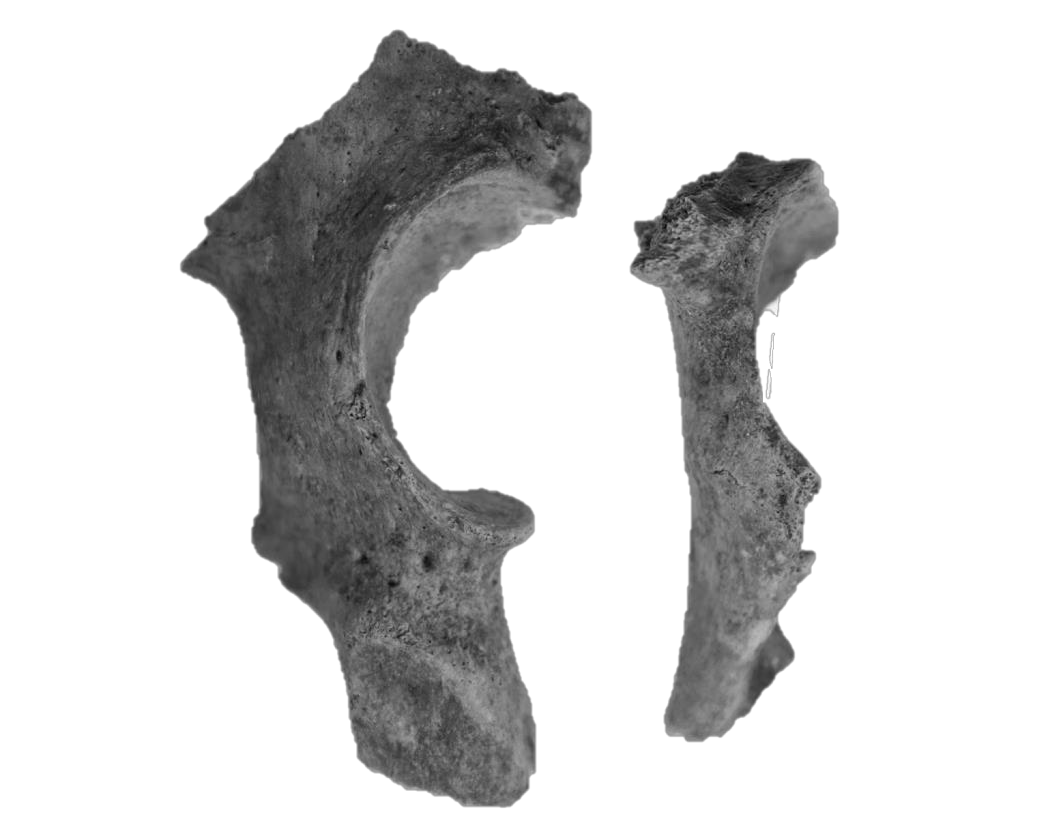


## **Supplemental Figure 6**. Duplicate right innominate found in Burial 8. Image reproduced from Kollmann (2021).


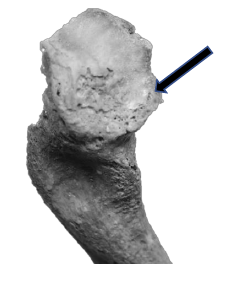


## **Supplemental Figure 7**. Eburnation of a vertebral facet of an upper rib from the Congregant in Burial 8. Image reproduced from Kollmann (2021).


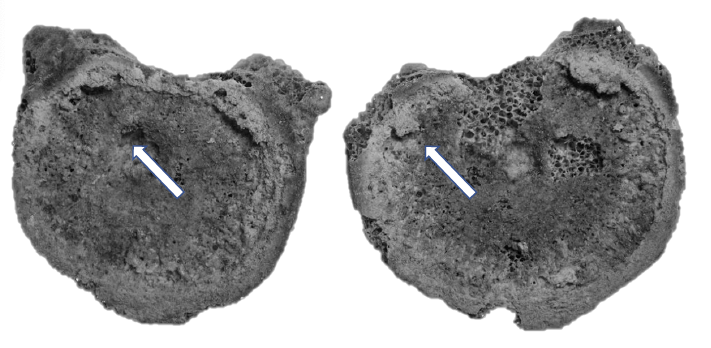


**Supplemental Figure 8**. Slight Schmorl’s depressions on the thoracic vertebrae from the Congregant in Burial 8. Image reproduced from Kollmann (2021).


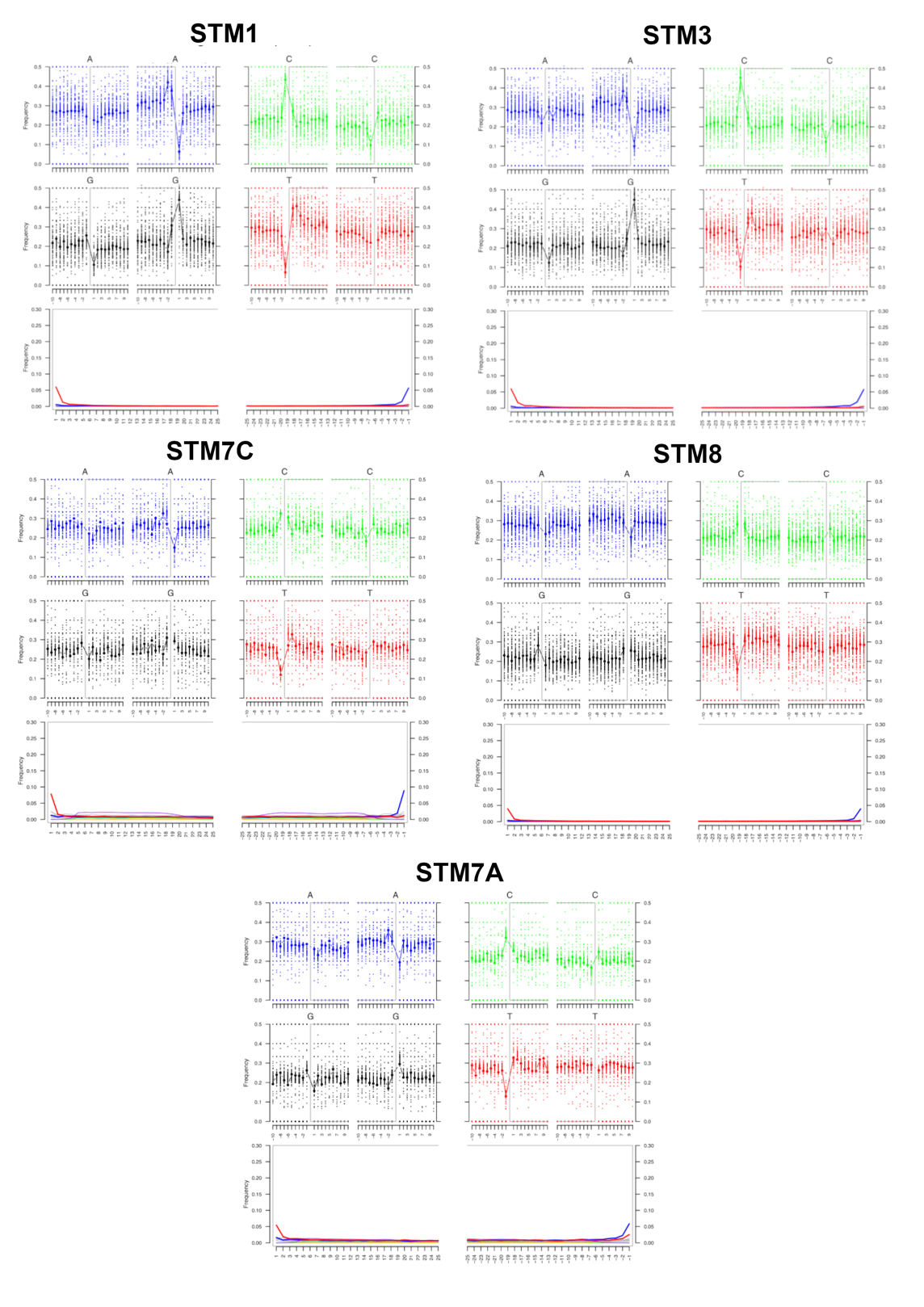


**Supplemental Figure 9.** Plots showing damage patterns for ancient DNA samples. Plots generated using MapDamage 2.0 (Jónsson et al. 2013). Notations for Burial Number and Sample ID are provided in Table 3.

 **Supplemental Figure 10.** Results from READ analysis, showing only high coverage samples analyzed using the MAX algorithm. Sample notations are as follows: A1 corresponds to STM1 (Crypt 1); A2 corresponds to STM3 (Crypt 3), A4 corresponds to STM8 (Burial 8).


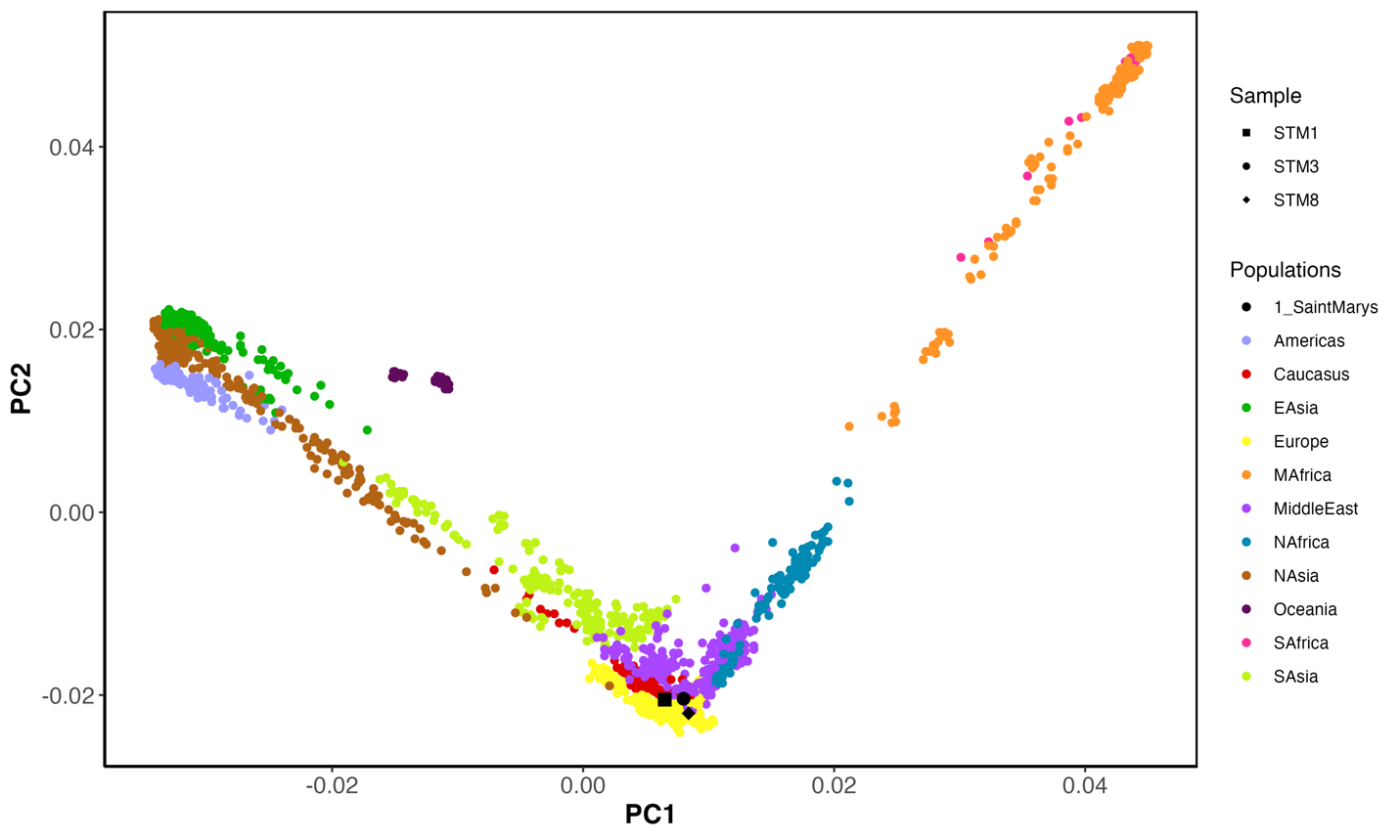


**Supplemental Figure 11**. SmartPCA of Congregants with the complete Human Origins reference panel. Notations for Burial Number and Sample ID are found in Table 3.

**
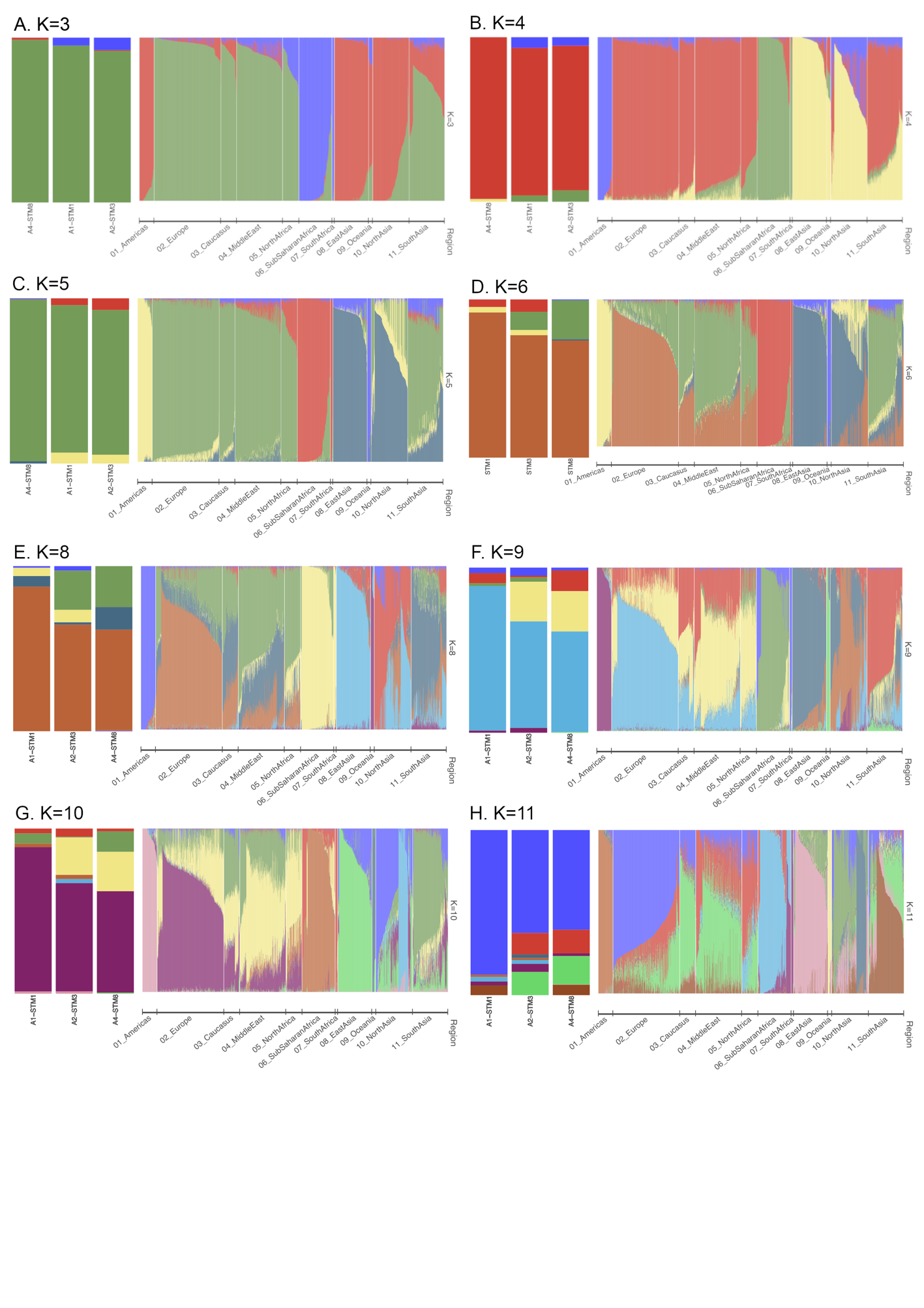
**

**Supplemental Figure 12**. ADMIXTURE analysis using the Human Origins reference panel. Notations for Burial Number and Sample ID are shown in Table 3.

**
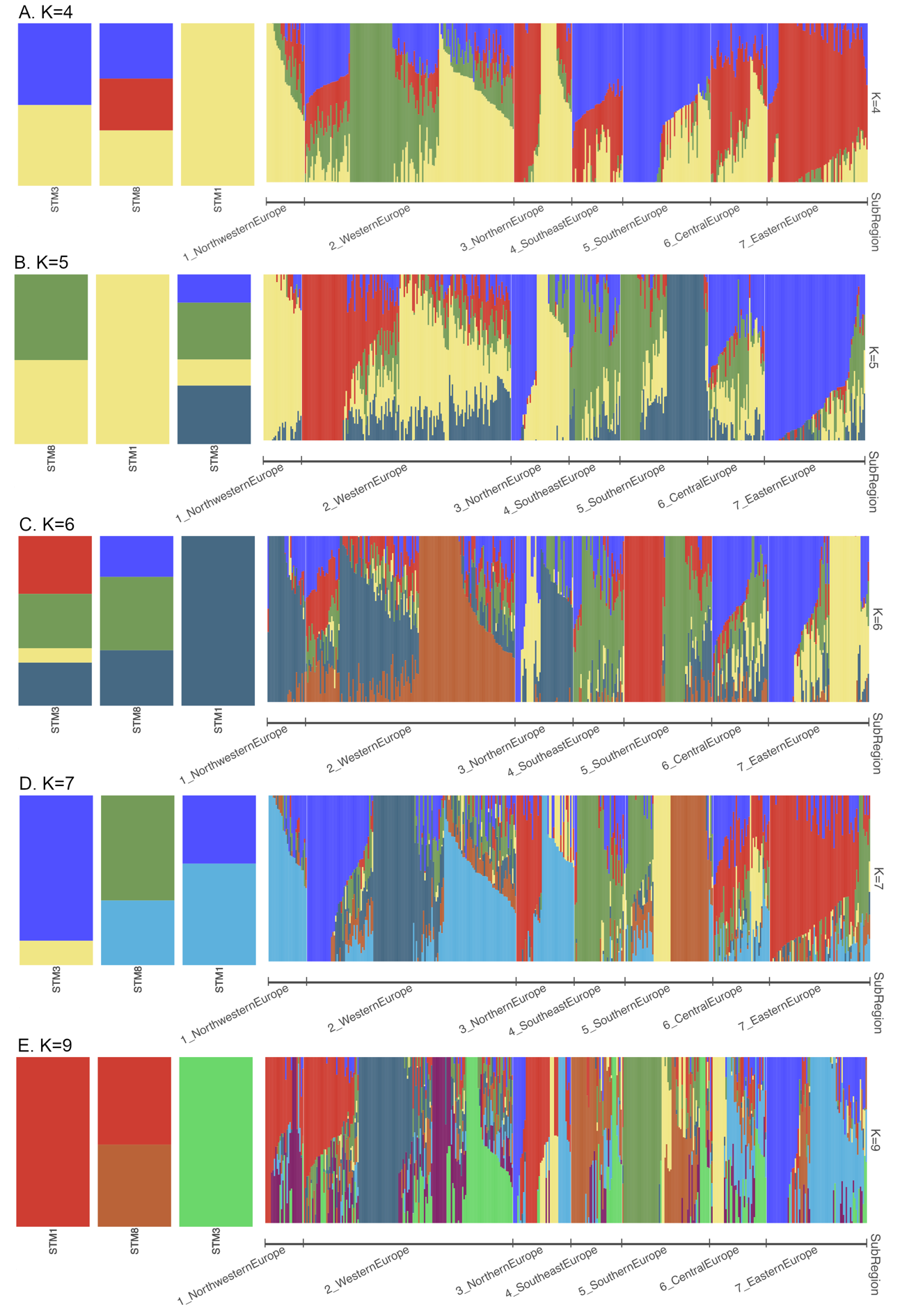
**

**Supplemental Figure 13**. ADMIXTURE analysis using only European populations from the Human Origins reference panel, organized by region. Notations for Burial Number and Sample ID appear in Table 3.

**
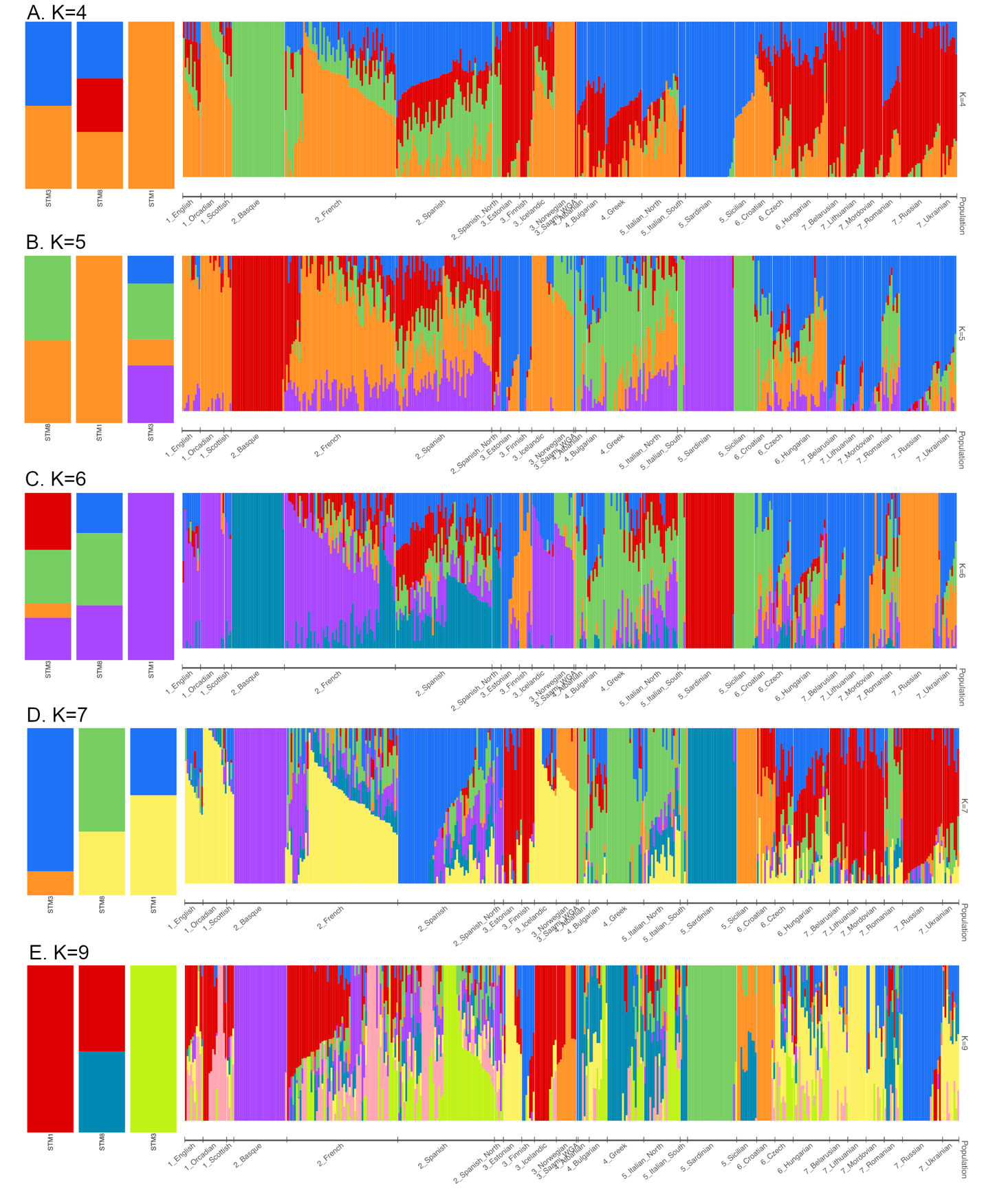
**

**Supplemental Figure 14**. ADMIXTURE analysis using only European populations from the Human Origins reference panel, organized by country. Notations for Burial Number and Sample ID are found in Table 3.

# Supplemental Tables.

| ID | Burial Location | DNA Sample | Preservation Notes | Sample Weight |
| --- | --- | --- | --- | --- |
| STM1 | Crypt 1 | Left Petrous | excellent preservation | 0.274 g |
| STM2 | Crypt 3 | Metatarsal (distal end) | good preservation | 0.245 g |
| STM3 | Crypt 3 | Right Petrous | good preservation | 0.240 g |
| STM7A | Burial 7 | Petrous Fragment | poor preservation | 0.200 g |
| STM7C | Burial 7 | Petrous Fragment | poor preservation | 0.241 g |
| STM8 | Burial 8 | Phalange (distal end) | excellent preservation | 0.243 g |

## **Supplemental Table 1**. DNA sample selection and preservation notes.

| Individual ID | PCR ID | Method 1 % Contamination | Method 1 SE | Method 2 % Contamination | Method 2 SE |
| --- | --- | --- | --- | --- | --- |
| STM1 | A1 | 0.31% | 0.003067861 | 0.35% | 0.003514932 |
| STM3 | A2 | 0.004103257% | 0.002001811 | 0.003278182 | 0.002045557 |
| STM7A | B3 | N/A | N/A | N/A | N/A |
| STM7C | A3 | N/A | N/A | N/A | N/A |
| STM 8 | A4 | N/A | N/A | N/A | N/A |

## **Supplemental Table 2**. X-chromosome contamination estimation.

| Individual ID | PCR ID | Number of Sequences | NchrY+ Nch rX | NchrY | R_y | SE | 95% CI | Genetic Sex Assign-ment |
| --- | --- | --- | --- | --- | --- | --- | --- | --- |
| STM1 | A1 | 5,387,927 | 153,459 | 16,450 | 0.1072 | 0.0008 | .1056-0.1087 | XY |
| STM3 | A2 | 18,457,958 | 544,674 | 58,281 | 0.107 | 0.0004 | .1062-0.1078 | XY |
| STM7A | B3 | 64,086 | 3,285 | 244 | 0.0743 | 0.0046 | .0653-0.0832 | Consistent with XY but not XX |
| STM7C | A3 | 135,367 | 5,818 | 286 | 0.0492 | 0.0028 | .0436-0.0547 | Not Assigned |
| STM8 | A4 | 19,633,878 | 1,024,123 | 6,447 | 0.0063 | 0.0001 | .0061-0.0064 | XX |

## **Supplemental Table 3**. Genetic sex assignment.

| Individual ID | PCR ID | Haplogroup | Haplogroup Marker | Total Number Of Reads | Number Of Valid Markers | QC  Score |
| --- | --- | --- | --- | --- | --- | --- |
| STM7A | B3 | N/A | N/A | 244 | 23 | N/A |
| STM7C | A3 | N/A | N/A | 286 | 32 | N/A |
| STM3 | A2 | R-FT8333 | Y177401 | 58281 | 12999 | 1 |
| STM1 | A1 | R-Y128530 | Y130082 | 16450 | 3294 | 1 |

## **Supplemental Table 4**. Y-chromosomal haplogroup assignment.
